# Advanced modeling and parameter estimation of PEM fuel cells using the *g*-function and self-adaptive differential evolution algorithm

**DOI:** 10.1038/s41598-025-27991-x

**Published:** 2025-12-22

**Authors:** Martin Ćalasan, Snežana Vujošević, Mihailo Micev, Shady H. E. Abdel Aleem, Brian Azzopardi

**Affiliations:** 1 Faculty of Electrical Engineering, University of Montenegro, Džordža Vašingtona, Podgorica, 81000 Montenegro; 2 Department of Electrical Engineering, Institute of Aviation Engineering and Technology, Giza, 12658 Egypt; 3 The Foundation for Innovation and Research – Malta (FiR.mt), Birkirkara, Malta; 4 Malta College of Arts, Science and Technology (MCAST), Paola, Malta; 5 Department of Systems and Control Engineering, Faculty of Engineering, The University of Malta (UM), Msida, Malta; 6 Azzopardi & Associates, Birkirkara, Malta

**Keywords:** PEM fuel cells, *g*-function, Lambert W function, Metaheuristic optimization, Parameter estimation, Renewable energy, Mathematical modeling, Energy storage, Fuel cells

## Abstract

Proton exchange membrane fuel cells (PEMFCs) have emerged as a promising technology due to their high efficiency, adaptability, and potential for integration into various applications, ranging from portable devices to large-scale power grids. A critical aspect of PEMFC research is the accurate modeling of its electrical characteristics. While traditional modeling approaches focus on the voltage-current relationship, there is a growing need to develop models that define current as a function of output voltage, particularly for control system applications. This study introduces a novel approach to PEMFC modeling using the *g*-function, a logarithmic transformation of the Lambert W function, which has been successfully applied in solar cell modeling. The *g*-function overcomes numerical limitations associated with the Lambert W function, ensuring greater stability and accuracy in computational applications. Additionally, this research proposes the self-adaptive differential evolution (SaDE) algorithm, a new metaheuristic optimization technique for estimating PEMFC parameters, addressing the need for robust and efficient parameter determination. To validate the proposed approach, a comprehensive comparative analysis is conducted against existing modeling and parameter estimation methods. Furthermore, sensitivity analysis is performed to assess the impact of parameter variations on model performance. The comparative evaluation across three different PEMFC systems (Ballard Mark V, BCS 500, and NedStack PS6) demonstrates consistent improvements, with RMSE reductions of up to 6.65% and SSE gains as high as 12.87%. These quantitative results highlight not only the enhanced accuracy but also the robustness and transferability of the proposed methodology across different fuel cell types. The results demonstrate that the proposed methodology offers improved numerical stability, enhanced accuracy, and efficient parameter estimation compared to conventional approaches. This study contributes to advancing PEMFC modeling techniques, providing a reliable framework for optimizing fuel cell performance and supporting their integration into sustainable energy systems. Overall, this study contributes a validated and versatile framework for advancing PEMFC modeling and optimization, supporting their integration into sustainable and real-world energy systems.

## Introduction

 Modern power systems are facing a series of complex challenges, including limited fossil fuel reserves, growing environmental concerns, and the unpredictability of global markets. These factors not only threaten long-term energy stability but also have a significant impact on environmental sustainability and the overall quality of life on the planet. In response to these challenges, there is an increasing focus on transitioning to renewable energy sources such as solar power, wind energy, biomass, and similar alternatives^1^.

However, these sources are not without limitations—their dependence on weather conditions, relatively low conversion efficiency, the need for significant spatial resources, and the inability to generate energy exactly when needed are among the major drawbacks. Another critical issue is the requirement for reliable energy storage systems, as current battery technologies are not yet sufficiently developed to support large-scale systems for long-term demand and supply balancing^2^.

In this context, fuel cells^3^, particularly proton exchange membrane fuel cells (PEMFCs)^4^, represent a promising technology, offering an innovative and efficient solution to the challenges of modern energy systems. Fuel cells are devices that directly convert the chemical energy of fuels, such as hydrogen, into electrical energy through electrochemical reactions, bypassing the combustion process. This technology enables a high conversion efficiency, typically ranging between 40% and 60% in standalone systems, while in combined heat and power (CHP) systems, efficiency can reach up to 90%. Such high efficiency not only allows for significant fuel savings but also reduces greenhouse gas emissions, making PEMFC technology a far more sustainable alternative compared to conventional energy generation methods.

Moreover, fuel cells exhibit high adaptability, as they can operate under a wide range of conditions while providing a stable and reliable energy source, regardless of external factors^5^. Their compact design and ability to be integrated into various systems—from small electronic devices to large-scale power grids—make them highly versatile, with applications spanning transportation, industry, and stationary energy systems. Another significant advantage is the potential use of renewable energy sources for hydrogen production, further reducing dependence on fossil fuels and enhancing the sustainability of energy generation.

Due to their exceptional efficiency, fuel savings, and environmental sustainability, fuel cells represent a key technology in transitioning toward more sustainable and efficient power systems. Consequently, these fuel cells have become the focus of extensive research, particularly in optimizing their performance and reliability^6–38^. In this context, sophisticated mathematical models based on equivalent electrical circuits are being developed, enabling detailed simulation and analysis of the key output characteristics of fuel cells. Among the various types of fuel cells, PEMFCs stand out as particularly promising for further development and widespread application. As a result, these fuel cells have become the subject of extensive research efforts. As part of these research initiatives, mathematical models based on equivalent electrical circuits are being developed to facilitate the simulation and analysis of key output characteristics of PEM fuel cells^6–45^.

Recent studies further confirm that, despite significant progress, open challenges still remain in the field of PEMFC system optimization. In study^39^, a biologically inspired algorithm, the Circulatory System-Based Optimization (CSBO), was proposed for accurate parameter identification of PEMFC models. It was validated on experimental data from commercial systems and demonstrated improved estimation accuracy. Study^40^ introduces the Pied Kingfisher Optimizer (PKO) metaheuristic, highlighting its robustness and capability to minimize the error between measured and simulated voltage values. In^41^, an enhanced differential evolution algorithm, Differential Evolution Ameliorated (DEA), was developed, enabling more reliable parameter estimation and emphasizing the importance of accurate identification for modeling, optimization, and fault detection. Finally^42^, presents a fuzzy-predictive control strategy for PEMFC systems and confirms its effectiveness through hardware-in-the-loop (HIL) testing, thereby underlining the necessity of algorithm validation under real operating conditions. These studies clearly indicate that a research gap still exists in balancing computational efficiency, accuracy, and experimental validation, which further justifies the contribution of the present work.

It is important to emphasize that, according to the available literature^6–12,14,15,17–42^, the mathematical modeling of PEM fuel cells is predominantly conducted by determining the voltage as a function of current, which represents the standard approach for analyzing the fundamental performance of the cell. This method provides insight into key operational characteristics, such as the polarization curve, as well as losses caused by activation, resistance, and concentration limitations.

However, it is crucial to develop models that enable the representation of current as a function of output voltage^13,16,43–45^. On one hand, this approach is important for mathematical modeling as it ensures the invertibility of the fuel cell model. On the other hand, it also holds significant practical relevance. Specifically, for a given voltage value, the corresponding current of the PEMFC can be accurately estimated. This is particularly important in fuel cell control systems, where the objective is to adjust the voltage at the cell terminals while simultaneously monitoring and estimating the current.

In the literature, there is a limited number of studies focusing on the current-voltage modeling of PEM fuel cells. Two main approaches can be identified in this domain. The first approach, proposed in^13,43–45^, is based on the application of the Lambert W function. The second approach, described in^16^, relies on iterative procedures that solve nonlinear equations, to which the current-voltage relationship can be reduced. Therefore, this field presents opportunities for further research and the development of novel methodologies aimed at improving existing modeling techniques.

This study is motivated by recent research on the numerical limitations of the Lambert W function. Specifically, based on the findings presented in^46–49^, which are grounded in the IEEE numerical standard IEEE 754–2008^50^, it has been demonstrated that the application of the Lambert W function is constrained by the value of its argument. More precisely, if the argument of the Lambert W function exceeds 10³²³, the function cannot be applied. Such large argument values frequently occur in the modeling of solar cells, particularly in describing the voltage-current dependence. To address this issue, both for simpler single-diode models and more complex two-diode and three-diode models, the use of the *g-function* (LogWright function) has been proposed. This function represents the logarithmic value of the Lambert W function. The key advantage of the *g*-function lies in the fact that its argument is equal to the logarithmic value of the argument of the Lambert W function. This transformation effectively eliminates the numerical limitations associated with modeling real-world problems, ensuring greater stability and accuracy in computational applications.

In the available literature, two main approaches can be identified for solving the *g*-function. The first is an analytical approach, described in^48^, which proposes the application of the *g*-STFT solution. However, the applicability of this solution is limited to a specific range of *g*-function arguments. The second, and most widely used, approach relies on iterative methods. In^49^, a highly efficient iterative method, referred to as *g*-IP, is proposed. Its main advantage is its independence from initial conditions. However, *g*-IP is limited to argument values greater than 1. On the other hand, the most commonly applied iterative procedure is based on Halley’s iterations, as described in^46,47^. Furthermore^46^, presents a procedure for defining the initial conditions of this iterative approach, making it applicable across all argument ranges of the *g*-function.

In this study, a novel approach to current-voltage characteristic modeling of PEMFC fuel cells will be proposed, based on the application of the *g*-function. Consequently, this research represents an original contribution to solving this scientific problem, leveraging existing knowledge on the potential applications of this function in solar cell modeling.

Regardless of whether PEMFC fuel cell modeling is conducted using the voltage-current or current-voltage relationship, these cells are characterized by a large number of parameters that define their performance. In the literature, metaheuristic algorithms are predominantly used for determining unknown parameters. The relevance of this topic is further supported by the fact that, in recent years, numerous new algorithms have emerged, with their application being proposed for solving this scientific problem.

Therefore, it is evident that testing the application and efficiency of new metaheuristic algorithms for parameter estimation remains a highly relevant research challenge. In this study, the self-adaptive differential evolution (SaDE) algorithm will be proposed for parameter estimation of PEMFC fuel cells. However, the key challenge of this research lies in comparing the efficiency of the proposed algorithm against existing literature approaches, an analysis that will be conducted as part of this study.

Based on the previous findings, the main contributions of this study are:

- The application of the *g*-function in the modeling of PEMFC fuel cells has been proposed.
- The SaDE algorithm has been proposed for the estimation of PEMFC fuel cell parameters.
- The efficiency of the proposed approach (model and algorithm) has been tested in comparison with the literary approaches.
- The sensitivity of the solution to parameter value variations has been tested.
- The applicability of the modeling approach has been tested for different operating conditions (pressure and temperature).

This paper is organized as follows: Sect. 2 presents the standard mathematical model of the equivalent circuit of a fuel cell, described through the relevant analytical relations. Section 3 explores the application of the *g*-function in modeling the current-voltage characteristics of PEMFC fuel cells. Section 4 introduces the SaDE optimization algorithm. Section 5 presents the application of the proposed SaDE algorithm to Ballard V 5 and BCS 500 fuel cells, along with a comparison of the results obtained from recent literature. Section 6 analyzes the effects of temperature and pressure variations on the obtained results, as well as sensitivity analysis. The conclusion summarizes the key findings and contributions of the study, highlighting the advantages of the proposed methodology and offering directions for future research.

## PEMFC and parameters estimation procedure

In this section, the first part presents the mathematical description of PEMFC fuel cells. The second part provides an overview of the parameter estimation process and defines the most commonly used criterion in this procedure.

### Mathematical description of PEMFC

This section presents the mathematical model of the PEM fuel cell. The mathematical representation of the PEMFC is based on Mann’s model, which has been developed to accurately depict polarization curves. Since the output voltage of a single fuel cell typically ranges from 0.9 V to 1.3 V, achieving the required voltage and current levels necessitates series, parallel, or combined configurations of multiple cells.

The mathematical expression for the stack output voltage is given by:

1 $${V_{stack}}={N_{cells}}\left( {{E_{Nernst}} - {V_{act}} - {V_\Omega } - {V_{conc}}} \right)$$

where *N*_*cells*_, *E*_*Nernst*_, *V*_*act*_, *V*_*Ω*_, and *V*_*conc*_ represent the number of series-connected fuel cells, the Nernst voltage of the cell, the activation overvoltage, the ohmic voltage drop per cell, and the concentration overvoltage, respectively.

The Nernst voltage, *E*_*Nernst*_ ​, can be described by the following equation:

2 $${E_{Nernst}}=1.229 - 0.85 \times {10^{ - 3}}\left( {{T_{fc}} - 298.15} \right)+4.3085 \times {10^{ - 5}} \times {T_{fc}} \times \log \left( {{P_{{H_2}}}\sqrt {{P_{{O_2}}}} } \right)$$

where *T*_*fc*_ is the operating temperature of the fuel cell (K), and *P*_*H*2_ and *P*_*O*2_ are the partial pressures of hydrogen and oxygen (atm), respectively. The activation overvoltage, *V*_*act*_, is given by the following equation:

3 $${V_{act}}= - \left[ {{\xi _1}+{\xi _2}{T_{fc}}+{\xi _3}{T_{fc}}\log \left( {{C_{{O_2}}}} \right)+{\xi _4}{T_{fc}}\log \left( {{I_{fc}}} \right)} \right]$$

$${C_{{O_2}}}=\left( {\frac{{{P_{{O_2}}}}}{{5.08 \cdot {{10}^6}}}} \right){e^{\frac{{498}}{{{T_{fc}}}}}}$$

where *I*_*fc*_ is the fuel cell current and represents a *ξ*_*1*_*- ξ*_*4*_ constant.

The ohmic voltage drop per cell is calculated using the following equation:

4 $${V_\Omega }={I_{fc}}\left( {{R_m}+{R_c}} \right)$$

where *R*_*m*_ represents the membrane resistance, and *R*_*c*_ denotes the contact resistance, both expressed in ohms.

The membrane resistance is given by the following expression:

5 $${R_m}=\frac{{{\rho _m}l}}{{{M_A}}}$$

so that;

6 $${\rho _m}=\frac{{181.6\left[ {1+0.03\left( {\frac{{{I_{fc}}}}{{{M_A}}}} \right)+0.062{{\left( {\frac{{{T_{fc}}}}{{303}}} \right)}^2}{{\left( {\frac{{{I_{fc}}}}{{{M_A}}}} \right)}^{2.5}}} \right]}}{{\left( {\lambda - 0.634 - 3\frac{{{I_{fc}}}}{{{M_A}}}} \right){e^{4.18 \cdot \frac{{{T_{fc}} - 303}}{{{T_{fc}}}}}}}}$$

where *ρ*_*m*_ represents the membrane resistivity, (Ω.cm), *l* denotes the membrane thickness (cm), *M*_*A*_ is the membrane surface area (cm²), and *λ* is an adjustable parameter.

The concentration overvoltage, *V*_*conc*_​, can be expressed by the following relation:

7 $${V_{conc}}= - \beta \log \left( {1 - \frac{J}{{{J_{\hbox{max} }}}}} \right)$$

where *β* represents the parametric coefficient, *J* denotes the current density (A/cm²), and *J*_max_ is the maximum current density (A/cm²).

The presented equations establish a formal mathematical framework for the comprehensive description and analysis of the physicochemical processes that define the operation of PEMFCs.

### Estimation of PEMFC parameters

During the estimation process, a fuel cell is predominantly observed by its voltage-current characteristic^6–12,14,15,17–42^, which defines the dependence of the cell’s output voltage on the load current. This characteristic enables the analysis of the cell’s performance, identification of loss factors, and determination of optimal operating conditions. Typically, the model is defined using seven key parameters (constants *ξ*_*1*_*- ξ*_*4*_, *λ*,* R*_*c*_ i *β*). Therefore, in the estimation process, it is essential to determine these parameters with the highest possible accuracy.

The accuracy of the estimation is verified by calculating standard statistical measures, specifically the root mean square error (RMSE) and the sum of squared errors (SSE). The mathematical background of these measures is given in Table 1.

**Table 1:** RMSE and SSE calculation.

Estimation criteria	Formula
Root mean square error - RMSE	$$\begin{gathered} RMS{E_V}=\sqrt {\frac{1}{{{N_m}}}\sum\limits_{{i=1}}^{{{N_m}}} {{{\left( {V_{i}^{{meas}} - V_{i}^{{calc}}} \right)}^2}} } \hfill \\ \hfill \\ \end{gathered}$$
Sum of squared errors - SSE	$SS{E_V}=\sum\limits_{{i=1}}^{{{N_m}}} {{{\left( {V_{i}^{{meas}} - V_{i}^{{calc}}} \right)}^2}}$

## Application of the *g*-function in modeling the current-voltage characteristics of PEMFCs

This chapter presents the application of the *g*-function in modeling PEMFC. However, a brief overview of this function is first provided, along with a comparison to the Lambert W function.

### Lambert W and *g*-functions

Lambert W function (or Lambert W) is a special function that solves equations of the form *x·e*^*x*^=*x*, where the variable *x* appears both in the base and the exponent^51–53^. This function is particularly useful in various fields of mathematics and physics, especially when solving equations analytically that involve the product of a quantity and its exponential function. Specifically, if we have the equation:

8 $$x \cdot {e^x}={k_1}$$

where *x* is the variable and ​ *k*_1_ is the argument, its solution is given by:

9 $$x=W\left( {{k_1}} \right)$$

The Lambert W function consists of two branches (the principal branch and the real-negative branch) when dealing with real values, while there are infinitely many branches in the case of complex values.

However, this function becomes numerically inapplicable when the argument value exceeds 10^323^^50^. In such cases, the application of the *g*-function has been proposed in^49^, which takes the following form:

10 $$g=\log \left( x \right)=\log \left( {{k_1}} \right) - x$$

where *α* = log(*k*_1_) is the argument of the *g*-function.

**Fig. 1:**
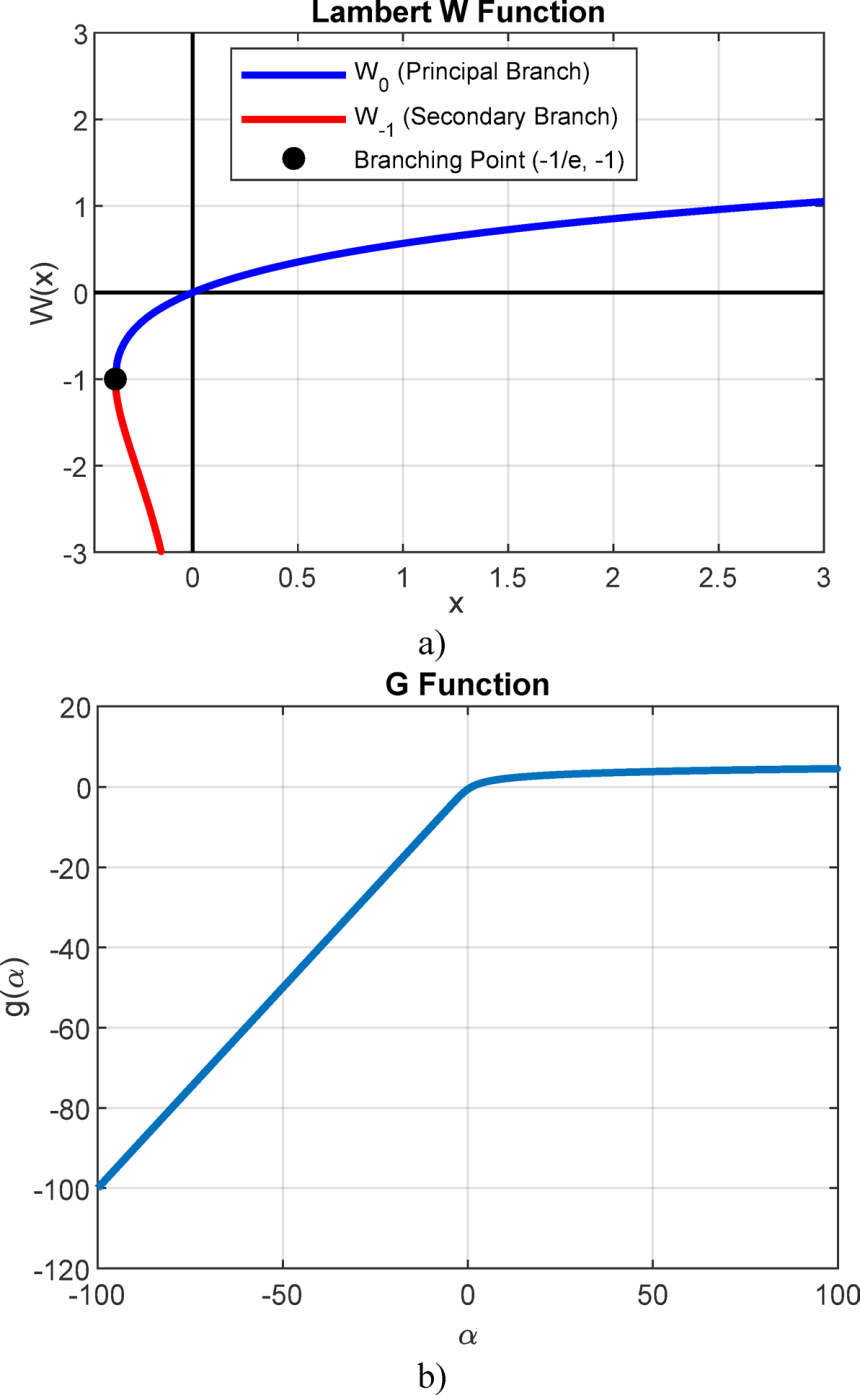
The graphical representation of the: (**a**) Lambert W function and (**b**) *g*-function.

The graphical representation of the Lambert W function and the *g*-function is shown in Fig. 1a,b.

### Current-voltage characteristics of the PEMFCs using the *g*-function

The fundamental voltage-current equation of the PEMFC can be transformed into the following form:

11 $$\begin{gathered} \frac{V}{N}=1.229 - 0.85 \times {10^{ - 3}}\left( {{T_{fc}} - 298.15} \right)+4.3085 \times {10^{ - 5}} \times {T_{fc}} \times \log \left( {{P_{{H_2}}}\sqrt {{P_{{O_2}}}} } \right) \hfill \\ {\text{ +}}{\xi _1}+{\xi _2}{T_{fc}}+{\xi _3}{T_{fc}}\log \left( {{C_{{O_2}}}} \right)+{\xi _4}{T_{fc}}\log \left( I \right) \hfill \\ {\text{ }} - I\left( {{R_c}+\frac{l}{{{M_A}}}\frac{{181.6\left[ {1+0.03\left( {\frac{I}{{{M_A}}}} \right)+0.062{{\left( {\frac{{{T_{fc}}}}{{303}}} \right)}^2}{{\left( {\frac{I}{{{M_A}}}} \right)}^{2.5}}} \right]}}{{\left( {\lambda - 0.634 - 3\frac{{{I_{fc}}}}{{{M_A}}}} \right){e^{4.18 \cdot \frac{{{T_{fc}} - 303}}{{{T_{fc}}}}}}}}} \right) \hfill \\ {\text{ +}}\beta \log \left( {1 - \frac{I}{{{M_A}{J_{\hbox{max} }}}}} \right) \hfill \\ \end{gathered}$$

By applying appropriate mathematical transformations, the preceding equation can be expressed in the following form^13,16,44^:

12 $${e^{{a_3}+{a_4}I+{a_5}{I^2}+{a_6}{I^{2.5}}}}={I^{ - {b_1}g}}{I^{{b_1}hI}}{\left( {1 - mI} \right)^{\beta \left( {g - hI} \right)}}$$

where:

$$E=1.229 - 0.85 \times {10^{ - 3}}\left( {{T_{fc}} - 298.15} \right)+4.3085 \times {10^{ - 5}} \times {T_{fc}} \times \log \left( {{P_{{H_2}}}\sqrt {{P_{{O_2}}}} } \right)$$

$$a=+{\xi _1}+{\xi _2}{T_{fc}}+{\xi _3}{T_{fc}}\log \left( {{C_{{O_2}}}} \right)$$

$$b={\xi _4}{T_{fc}}$$, $$c={R_c}$$, $$d=181.6\frac{l}{{{M_A}}}$$, $$e=0.03/{M_A}$$, $$f=0.062{\left( {\frac{{{T_{fc}}}}{{303}}} \right)^2}{\left( {\frac{1}{{{M_A}}}} \right)^{2.5}}$$,

13 $$g = \left( {\lambda - 0.634} \right)e^{{4.18 \cdot \frac{{T_{{fc}} - 303}}{{T_{{fc}} }}}} ,\,h = \frac{3}{{M_{A} }}e^{{4.18 \cdot \frac{{T_{{fc}} - 303}}{{T_{{fc}} }}}} ,\,m = \frac{1}{{M_{A} J_{{\max }} }}$$

$$\begin{gathered} {a_3}={a_2}g \hfill \\ {a_4}= - {a_2}h+cg+d \hfill \\ {a_5}= - ch+de \hfill \\ {a_6}=df \hfill \\ {b_1}= - b \hfill \\ \end{gathered}$$

After certain mathematical formulations, the preceding equation can be rewritten in the following *g*-function form:

14 $$\log \left( \theta \right)+\theta =\Upsilon$$

where:

15 $$\Upsilon =\log \left( {\frac{{{a_4}}}{{{b_1}g}}} \right) - \frac{1}{{{b_1}g}}\left[ {{a_3}+{a_5}{I^2}+{a_6}{I^{2.5}} - {b_1}hI\log \left( I \right) - \beta \left( {g - hI} \right)\log \left( {1 - mI} \right)} \right]$$

and

16 $$\theta =\frac{{{a_4}I}}{{{b_1}g}}$$

In other words, the solution of (14)​, which represents the *g*-function for a given value of the variable$\Upsilon$, provides the value of the variable $\theta$from which the fuel cell current can be easily determined as:

17 $$I=\frac{{{b_1}g}}{{{a_4}}}\theta$$

However, the variable$\Upsilon$ is a complex function that also includes the fuel cell current as one of its terms. Therefore, an iterative procedure must be applied to determine the current. In this study, the iterative procedure is based on the application of the iterative *g*-function. Namely, for a given initial value of the fuel cell current (denoted as ^(0)^*I*), an initial value of the variable $\Upsilon$ (denoted as $^{{\left( 0 \right)}}\Upsilon$) is determined (see Eq. (15)). Then, the *g*-function Eq. (14) is solved.

To solve the g-function, one of the methods described in the literature^46–49^ can be utilized. Once the *g*-function is solved, i.e., once the value of the variable $\theta$is determined, a new value of the fuel cell current (denoted as ^(1)^*I*), can be calculated. In the next step, a new value of the variable $\Upsilon$, denoted as $^{{\left( 1 \right)}}\Upsilon$ is computed, for this updated current value. If the difference between the initial state of the variable denoted as $^{{\left( 0 \right)}}\Upsilon$ and the new state denoted as $^{{\left( 1 \right)}}\Upsilon$ is smaller than a predefined convergence criterion (e.g., on the order of 10^–10^), the iterative procedure is terminated. However, if the criterion is not met, the newly obtained current value ^(1)^*I* is taken as the initial current value ^(0)^*I*, and the entire procedure is repeated.

However, similar to the application of the Lambert W equation in modeling the current of PEMFC cells, the proposed iterative procedure may also lead to the appearance of complex numbers as a result of the current value exceeding 1/*m*. Namely, from Eq. (15) it is evident that if the current exceeds 1/*m*, the situation arises where the logarithm of a negative number is required. For such cases, it is necessary to employ an alternative iterative procedure, such as the Halley iterative method or the original or modified Newton method.

## Self-adaptive differential evolution (SaDE) algorithm

Self-adaptive differential evolution (SaDE) is an advanced variant of the classical differential evolution (DE) algorithm, designed for solving global optimization problems^54^. Although the efficiency and success of the DE algorithm have been confirmed across various fields, the parameters and learning strategies used within the algorithm are problem-dependent. The SaDE algorithm was first proposed in^54^, and unlike the standard DE algorithm, the selection of the mutation strategy and the two control parameters, the mutation scaling factor (*F*) and the crossover probability (*CR*) do not need to be predefined. Therefore, the main advantage of the SaDE algorithm compared to the standard DE lies in its ability to automatically adjust the control parameters during the optimization process, thereby improving its efficiency and robustness in solving complex problems. Throughout the evolutionary process, the appropriate learning strategy and parameter settings gradually adapt on their own based on the experience gained during the search.

### Differential evolution algorithm

Differential evolution is an optimization algorithm that operates on a population of candidate solutions, *NP*, within an *n*-dimensional search space. This population consists of *NP* vectors in the form *X*_*i*_ = (*x*_*i*1_,…, *x*_*i*n_), where *i* = 1, …, *NP*, and each vector represents a potential solution to the problem. To ensure an effective exploration, the initial population should randomly cover the entire search space. Each component of the vector is initialized using a random process following a uniform distribution within the predefined lower (*x*_*l*_) and upper (*x*_*u*_) bounds.

The algorithm evolves the population over successive generations. In each generation *G*, the DE algorithm applies mutation and crossover to generate a trial vector *U****i***_,*G*_ for each target vector *Xi*_,*G*_ in the current population. The goal of this process is to produce improved solutions through the iterative refinement of the population.

#### Mutation procedure

**Fig. 2:**
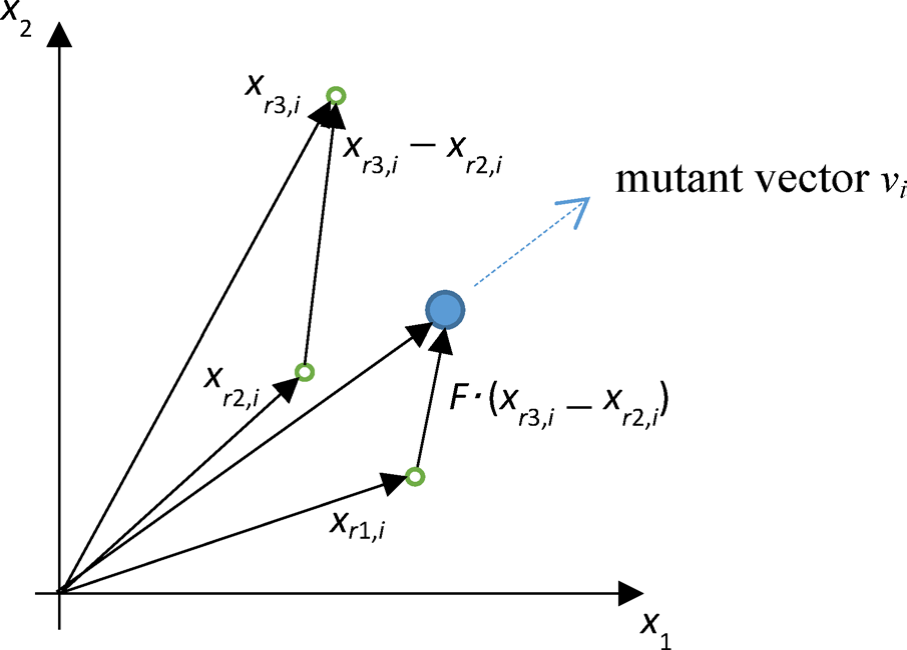
Graphical representation of mutation in the DE algorithm.

For each target vector X*i*_,*G*_ in generation *G*, the corresponding mutant vector *V*_*i, G*_=(*v*_1*i*,G_,…,*v*_n*i*,G_) can be generated using one of the following five strategies:

1. DE/rand/1: $${V_{i,G}}={X_{{r_1},G}}+F\left( {{X_{{r_2},G}} - {X_{{r_3},G}}} \right)$$
2. DE/best/1: $${V_{i,G}}={X_{best,G}}+F\left( {{X_{{r_1},G}} - {X_{{r_2},G}}} \right)$$
3. DE/current-to-best/1: ${V_{i,G}}={X_{i,G}}+F\left( {{X_{best,G}} - {X_{i,G}}} \right)+F\left( {{X_{{r_1},G}} - {X_{{r_2},G}}} \right)$
4. DE/best/2: $${V_{i,G}}={X_{best,G}}+F\left( {{X_{{r_1},G}} - {X_{{r_2},G}}} \right)+F\left( {{X_{{r_3},G}} - {X_{{r_4},G}}} \right)$$
5. DE/rand/2: $${V_{i,G}}={X_{{r_1},G}}+F\left( {{X_{{r_2},G}} - {X_{{r_3},G}}} \right)+F\left( {{X_{{r_4},G}} - {X_{{r_5},G}}} \right)$$

where the indices *r*_1_-*r*_5_ are randomly selected and mutually distinct numbers from the range [1, *NP*], which are also different from the index of the current vector *i*. The factor *F* is a scaling parameter within the range [0, 2], while *X*_*best,G*_ denotes the vector with the best objective function value in the population of generation *G*. The mutation procedure of DE algorithm is graphically presented in Fig. 2.

#### Crossover operation

After the mutation phase, the crossover operation is applied between the generated mutant vector *V*_*i, G*_ and its corresponding target vector *X*_*i, G*_ to form the trial vector *U*_*i, G*_=(*u*_1*i*,G_,…,*u*_n*i*,G_):

18 $$u_{{j,i,G}} = \left\{ {\begin{array}{*{20}c} {v_{{j,i,G}} ,{\mkern 1mu} if\left( {rand_{j} \left[ {0,1} \right] \le CR} \right)or\left( {j = j_{{rand}} } \right)} \\ {x_{{j,i,G}} ,{\mkern 1mu} {\mkern 1mu} {\mkern 1mu} {\mkern 1mu} {\mkern 1mu} {\mkern 1mu} otherwise} \\ \end{array} } \right.\,\,\,j = 1,2, \ldots ,n$$

where *CR* is the crossover constant within the range [0, 1), and *j*_*rand*_ is a randomly selected index from the range [1, *NP*], ensuring that the trial vector *U*_*i, G*_ differs from the target vector *X*_*i, G*_ in at least one component.

#### Selection operation

If any value in the trial vector exceeds the predefined parameter bounds, it is randomly reinitialized within the allowed range. After that, the objective function values of all trial vectors are calculated.

Selection is then performed as follows:

- If the objective function value of the trial vector is better than or equal to that of the target vector (for a minimization problem), the trial vector replaces the target vector and becomes part of the next generation’s population.
- Otherwise, the target vector remains in the population.

This can be mathematically expressed as:

19 $$X_{{i,G + 1}} = \left\{ {\begin{array}{*{20}c} {U_{{i,G}} ,\,if\,f\left( {U_{{i,G}} } \right) < f\left( {X_{{i,G}} } \right)} \\ {X_{{i,G}} ,\,\,\,\,\,\,\,\,\,\,\,\,\,\,\,otherwise} \\ \end{array} } \right.$$

These three phases are repeated until a predefined stopping criterion is satisfied.

### Implementation of the SaDE algorithm

In order to improve the performance of the DE algorithm for a specific problem, all five mutation strategies must be tested in the classical DE, and the key parameters *CR*, *F*, and *NP* must be carefully tuned. Moreover, during different phases of the evolution process, it may be desirable to apply different strategies, as some are more suitable for global exploration, while others are better for local optimization.

To address these challenges, a new algorithm, SaDE, has been proposed, which automatically adapts the learning strategies and parameter values during the evolutionary process.

#### Adaptation of the learning strategy

The goal is to probabilistically select the learning strategy rather than predefine it in advance. Therefore, two strategies are considered as candidates:

1. rand/l/bin $${V_{i,G}}={X_{{r_1},G}}+F\left( {{X_{{r_2},G}} - {X_{{r_3},G}}} \right)$$.
2. current to best/2/bin ${V_{i,G}}={X_{i,G}}+F\left( {{X_{best,G}} - {X_{i,G}}} \right)+F\left( {{X_{{r_1},G}} - {X_{{r_2},G}}} \right)$.

The rand/1/bin strategy is used because it provides good diversity, while the current-to-best/2/bin strategy enables fast convergence. If the probability of applying the rand/1/bin strategy to an individual in the current population is denoted as *p*_1_, it follows that the second strategy is applied with probability *p*_2_ = 1- *p*_1_. The initial probabilities of applying each strategy are equal (*p*_1_ = *p*_2_ = 0.5).

For a population of size *NP*, a vector of size *NP* is randomly generated, where each element has a value drawn from a uniform distribution in the range [0,1]. If the value of the *j*-th element of the vector is less than or equal to *p*_1_, the rand/1/bin strategy is applied to the *j*-th individual in the current population. Otherwise, the current-to-best/2/bin strategy is used. After evaluating all newly generated trial vectors, the number of vectors that successfully advanced to the next generation using the rand/1/bin and current-to-best/2/bin strategies is recorded as *ns*₁ and *ns*₂, respectively. Additionally, the number of discarded vectors generated by the same strategies is recorded as *nf*₁ and *nf*₂. These two values are accumulated over a predefined number of generations, referred to as the “learning period.”

#### Adaptation of strategy probabilities

After each learning period:

- The number of successful trial vectors generated by each strategy denoted as *ns*₁ and *ns*₂, is recorded.
- The number of unsuccessful trial vectors discarded during the selection process denoted as *nf*₁ and *nf*₂, is also recorded.
- Based on these values, the probabilities of applying each strategy are updated using the following equations:

20 $$\begin{gathered} {p_1}=\frac{{n{s_1}\left( {n{s_2}+n{f_2}} \right)}}{{n{s_1}\left( {n{s_2}+n{f_2}} \right)+n{s_2}\left( {n{s_1}+n{f_1}} \right)}}, \hfill \\ \hfill \\ {p_2}=1 - {p_1} \hfill \\ \end{gathered}$$

The first equation represents the success rate of trial vectors generated using the rand/1/bin strategy relative to the total success of trial vectors produced by both strategies during the learning period. Based on this, the probabilities of applying these strategies are updated at the end of the learning period, while the counters are reset. This adaptation process can gradually develop the most suitable learning strategy for different evolutionary phases, dynamically adjusting to the characteristics of the problem being addressed.

#### The population size *NP*, the mutation scaling factor *F*, and the crossover probability *CR*

In the original DE algorithm, the three key control parameters *CR*, *F*, and *NP* are closely related to the problem under consideration.

In the SaDE algorithm, only the population size *NP* is left as a user-defined value to enable solving problems of different complexities and dimensionalities.

The mutation factor *F* is predominantly associated with the convergence speed. In the SaDE algorithm, the scaling factor *F* is not fixed but is randomly generated from a normal distribution with:

- A mean value of 0.5.
- A standard deviation of 0.3.
- A value range of (0,2]

In this way, by combining small and large values of *F*, the algorithm can simultaneously explore and exploit the solution space. Mutant vectors generated using this method achieve a better balance between diversity and convergence, increasing the chances of finding an optimal solution during evolution.

The crossover probability parameter *CR* plays a crucial role in the original DE algorithm. Since it is typically confined to a very narrow range, its optimal tuning can be highly challenging. An inappropriate value can significantly impact the algorithm’s performance by either slowing down convergence or reducing solution diversity.

In the SaDE algorithm, the adjustment of *CR* is performed automatically based on successful mutations during evolution, ensuring dynamic optimization of the parameter following the characteristics of the problem. This approach enhances flexibility and maintains a balance between exploration and exploitation of the search space, ultimately improving the overall performance of the algorithm.

The values that CR can take range from [0.1, 1], with an initial value set at 0.5, which is updated using a normal distribution with a mean value of *CR*_m_ and a standard deviation of 0.1. This adaptive approach ensures algorithm stability while simultaneously providing sufficient variability during optimization.

**Fig. 3:**
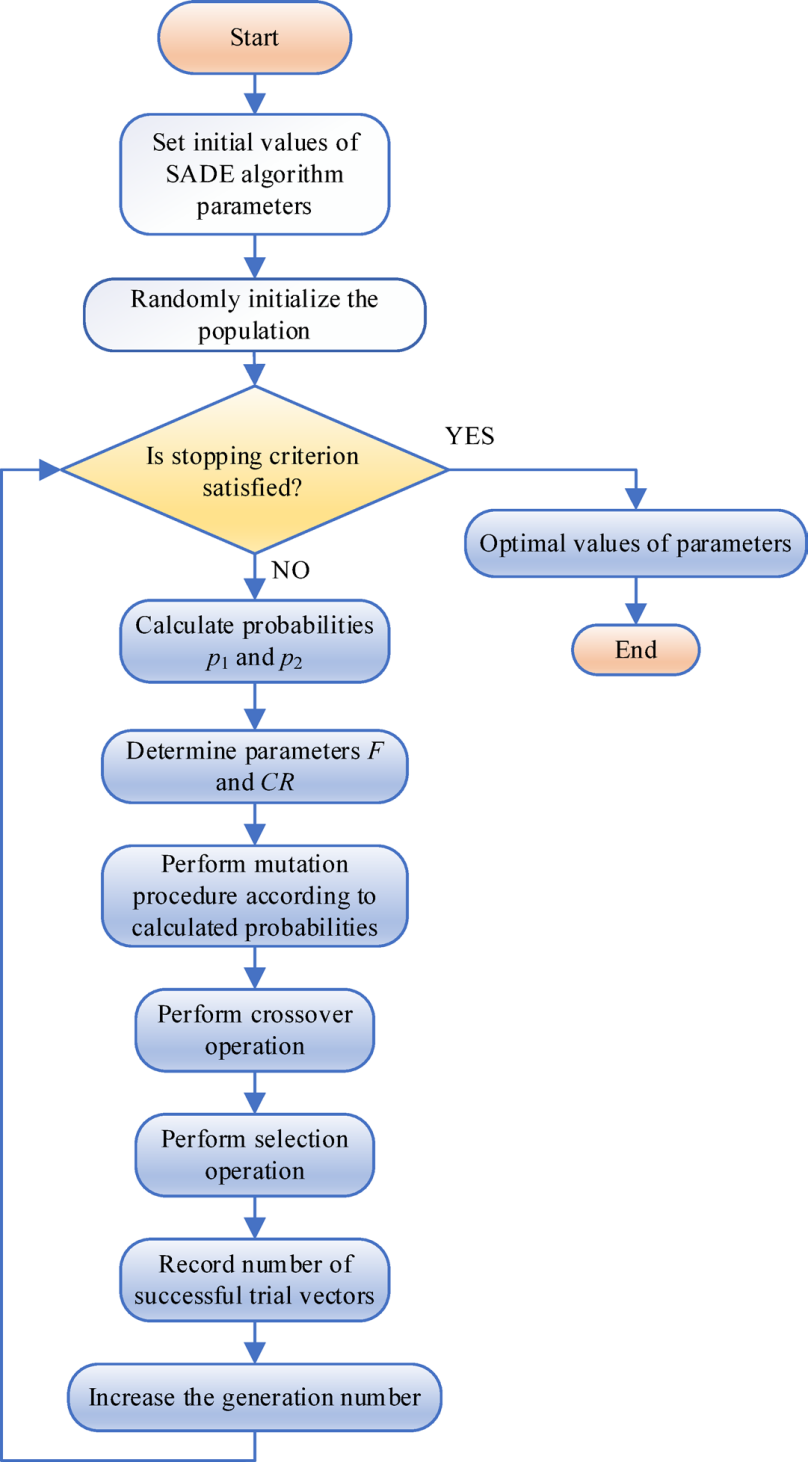
Algorithm flowchart.

In order to provide a visual representation of the proposed algorithm, the appropriate flowchart is depicted in Fig. 3.

## Numerical results

In this section, the efficiency of the proposed method for determining the parameters of the output current-voltage characteristics of fuel cells is evaluated. The testing includes three widely used types of PEMFC fuel cells: Ballard-Mark-V (5 kW), BCS (500 W), and NedSTack PS6 (6 kW). For computation and estimation, the SaDE algorithm was employed. The detailed specifications and experimental polarization data of the three commercial PEM fuel cell stacks (Ballard Mark, BCS, and NedStack PS6) used for model validation are provided in the Appendix. The detailed specifications and experimental polarization data of the three commercial PEM fuel cell stacks (Ballard Mark, BCS, and NedStack PS6) used for model validation are provided in Appendix 1.

### BALLARD PEMFC

This subsection consists of four parts. In the first part, a comparison of numerous methods from the literature is presented in terms of RMSE. The second part provides a comparison of the convergence characteristics of several optimization algorithms. In the third part, the results of statistical tests are presented, while the fourth part includes a discussion on the computational complexity of the proposed algorithm.

#### RMSE comparisons

Table 2 presents data from the latest literature for 24 different methods, as well as the results obtained using the SaDE algorithm and the proposed calculation method for the Ballard fuel cell. In this goal, we proposed six different variants of PEMFC parameters. Each variant differs in the optimization boundary values of the key parameters *λ* and *R*_c_. By combining different values of membrane hydration level (*λ*) and cell resistance (*R*_c_) throughout the optimization process, the following variants were obtained:

- Variant 1 – Reference configuration, with *λ* = 23 and *R*_c_ = 0.1 mΩ.
- Variant 2 – Slightly increased membrane hydration, with *λ* = 23.5, while *R*_c_ remains unchanged (0.1 mΩ).
- Variant 3 – Further increased membrane hydration, with *λ* = 24, while keeping fixed at *R*_c_ = 0.1 mΩ.
- Variant 4 – Reduced resistance, with *R*_c_ = 0.09 mΩ, while *λ* remains at the reference value (23).
- Variant 5 – Combination of increased hydration and reduced cell resistance, with *λ* = 23.5 and *R*_c_ = 0.09 mΩ.
- Variant 6 – Best optimized configuration, with *λ* = 24 and *R*_c_ = 0.09 mΩ.

These variations demonstrate how changes in membrane hydration and cell resistance affect the model accuracy and the output performance of the fuel cell.

**Fig. 4:**
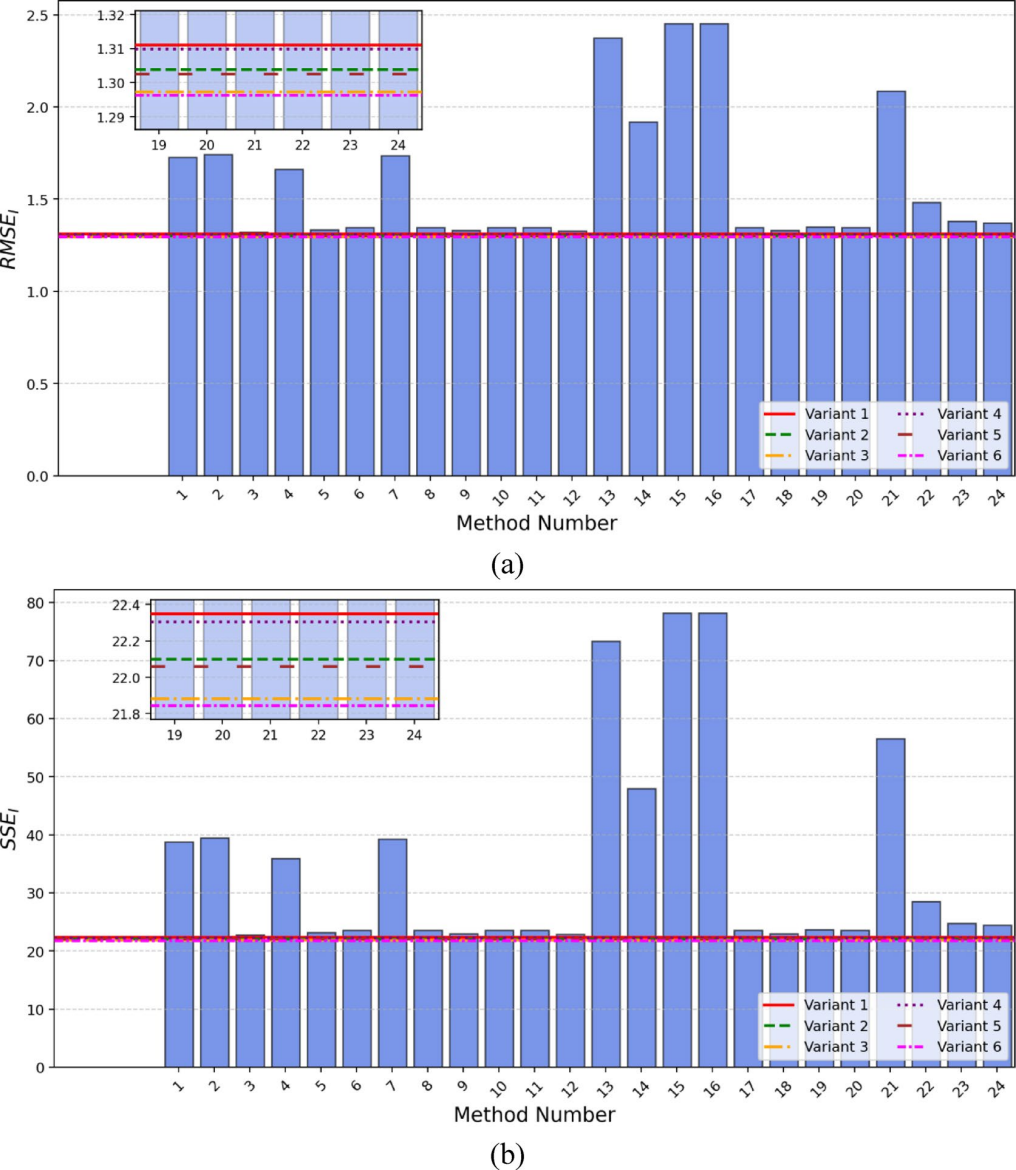
BALLARD PEMFC: (**a**) RMSE and (**b**) SSE comparisons.

Table 2 also presents the corresponding RMSE_I_ (root mean square error of current) and SSE_I_ (sum of squared errors of current) values as a metric for model error assessment. The comparison of RMSE and SSE for this PEMFC is depicted in Fig. 4a clearly shows that the highest RMSE values are associated with methods 15, 16, 13, 21, and 14, respectively. A similar trend is observed in Fig. 4b, which presents the SSE calculation, confirming that these methods demonstrate relatively higher error values within the analyzed set.

**Table 2:** Values of parameters obtained by applying the mentioned literature approaches - Ballard mark V fuel cell.

*n*	L	Algorithms	Journal	ξ_1_ (V)	ξ_2_ ·10 − 3 (V/K)	ξ_3_ · 10 − 5 (V/K)	ξ_4_· 10 − 5 (V/K)	λ	*R*_c_ (mΩ)	β (V)	RMSE_I_(A)	SSEE_I_ (A^2^)
1	^6^	KOA	Scient. Reports	-0.8814	3.1030	7.0050	-16.3984	23.0000	0.1000	0.0136	1.7272593504	38.7845232260
2	^6^	PSA	Scient. Reports	-1.0635	3.3482	4.9959	-16.2830	22.9999	0.1000	0.0136	1.7434607348	39.5155193387
3	^7^	DTBA	Scient. Reports	-0.8952	2.9699	5.8812	-15.6956	23.0000	0.0175	0.0136	1.3230099012	22.7546175832
4	^8^	EBOA	PeerJ. Comput. Sci.	-1.1897	3.6653	4.7175	-15.7171	23.0000	0.0141	0.0146	1.6622501501	35.9199822987
5	^9^	ARA 25	Sustainability	-1.1589	3.5208	4.0526	-16.7251	23.9900*	0.1000	0.0159	1.3342775056	23.1438540064
6	^10^	PFA48	Energy Conv. Manag.	-1.1997	3.9579	6.3901	-16.2830	23.0000	0.1000	0.0136	1.3458873794	23.5483668944
7	^11^	HBA46	Energy Conv. Manag.	-1.1997	4.3345	9.2069	-16.2830	23.0000	0.1000	0.0136	1.7376989990	39.2547715446
8	^12^	WOA33	Energy Conv. Manag.	-1.1978	4.4183	9.7214	-16.273	23	0.1002	0.0136	1.3455487331	23.5365181109
9	^13^	ERWCA 22	Energy	-0.8548	3.3043	8.8427	-16.7251	24.0000*	0.1000	0.0159	1.3305635460	23.0151915493
10	^14^	ABCDEA23	Energy	-1.1956	4.2189	8.3404	-16.2830	23.0000	0.1000	0.0136	1.3459605424	23.5509271621
11	^15^	ETSA30	Energy	-0.8534	2.5591	3.6100	-16.2868	23.0000	0.1000	0.0136	1.3462363400	23.5605796808
12	^16^	OOA-COA	Energy	-1.199826697	4.030256041	6.73528223	-17.4311767	24.994201423	0.09	0.017	1.3267670843	22.8840416478
13	^17^	-	Energy	-1.1991	3.6858	5.3268	-11.103	21.4356	0.62405	0.0158	2.3754286221	73.3545948030
14	^18^	LSA37	Energies	-1.0624	3.5970	6.6538	-16.4925	23.0000	0.1030	0.0188	1.9207881892	47.9625544810
15	^19^	HGSA33	Energies	-0.9910	3.7000	9.1000	-16.3500	22.8700	0.1000	0.0135	2.4524031574	78.1856562035
16	^20^	NNA49	Int. J. Energy Res.	-0.9800	3.6946	9.0871	-16.3000	23.0000	0.1000	0.0136	2.4524031574	78.1856562035
17	^21^	MRFA47	Int. J. Energy Res.	-1.0898	3.8249	7.7306	-16.2830	23.0000	0.1000	0.0136	1.3458631423	23.5475187714
18	^22^	MPA30	Ain Shams Eng. J.	-1.0793	3.8868	8.3282	-16.7251	24	0.1	0.0159	1.3311052606	23.0339357924
19	^23^	GOA34	IET Renew. Pow. Gen	-0.853	3.417	9.8	-15.95	22.84	0.1	0.0136	1.3497467371	23.6836113061
20	^24^	CSA26	Electron	-1.181342405	3.569096	3.9929	-16.2830	23	0.1	0.0136	1.3459153748	23.5493465496
21	^24^	NNA26	Electron	-1.159421713	3.604278	4.926	-14.7034	23	0.1	0.0136144	2.0860743094	56.5721783164
22	^24^	GWO26	Electron	-0.904692414	2.870928	5.2237	-13.0626	23	0.50941	0.0136437	1.4813673912	28.5278415202
23	^24^	SCA26	Electron	-0.891394545	2.651533	3.6	-15.6624	23	0.17819	0.0136	1.3792726233	24.7311086020
24	^25^	L-AHA	Measurem.	-0.9448	2.8351	3.6000	-16.524	23.0000	0.1	0.0136	1.3711256364	24.4398116403
25	SaDE - Variant 1			-0.924324296179	3.4820136837676	9.0314732695386	-14.984808618621	23	0.1	0.01508348902	1.3112210023	22.3509067193
26	SaDE - Variant 2			-1.182050094695	4.3117636929434	9.5592086512130	-15.188074992647	23.5	0.1	0.01650099089	1.3038561971	22.1005327753
27	SaDE - Variant 3			-1.065294664991	3.5557654256650	6.5576333912740	-15.380459082034	24	0.1	0.01785143196	1.2974379414	21.8834877532
28	SaDE - Variant 4			-1.017898033388	3.0670797663330	4.1078927264736	-15.036461971605	23	0.09	0.01520755569	1.3099133968	22.3063503925
29	SaDE - Variant 5			-0.995236910761	3.4202750479780	7.0725747927280	-15.239557875657	23.5	0.09	0.01662647090	1.3026447073	22.0594820349
30	SaDE - Variant 6			-1.096006810748	3.2695796657657	3.8612399628978	-15.447876557123	24	0.09	0.01793241467	1.2963193226	21.8457692214

The analysis of the results indicates that, among the methods from the literature, the DTBA method (method No. 3 in Table 2 achieved the best result, with the lowest RMSE_I_ value of 1.32301. However, all six variations implemented using the SaDE algorithm and the proposed calculation model exhibited lower error values compared to the methods from the literature. The best result was achieved with Variant 6, which had an RMSE_I_ value of 1.29631.

When it comes to SSE_I_, the best result among the methods from the literature was achieved using the DTBA method, with a value of 22.75461. Nevertheless, in this aspect as well, all six variations implemented using the SaDE algorithm and the proposed calculation model exhibited lower error values compared to the methods from the literature. The best result was obtained with variant 6, which had an SSE_I_ value of 21.84576.

**Fig. 5:**
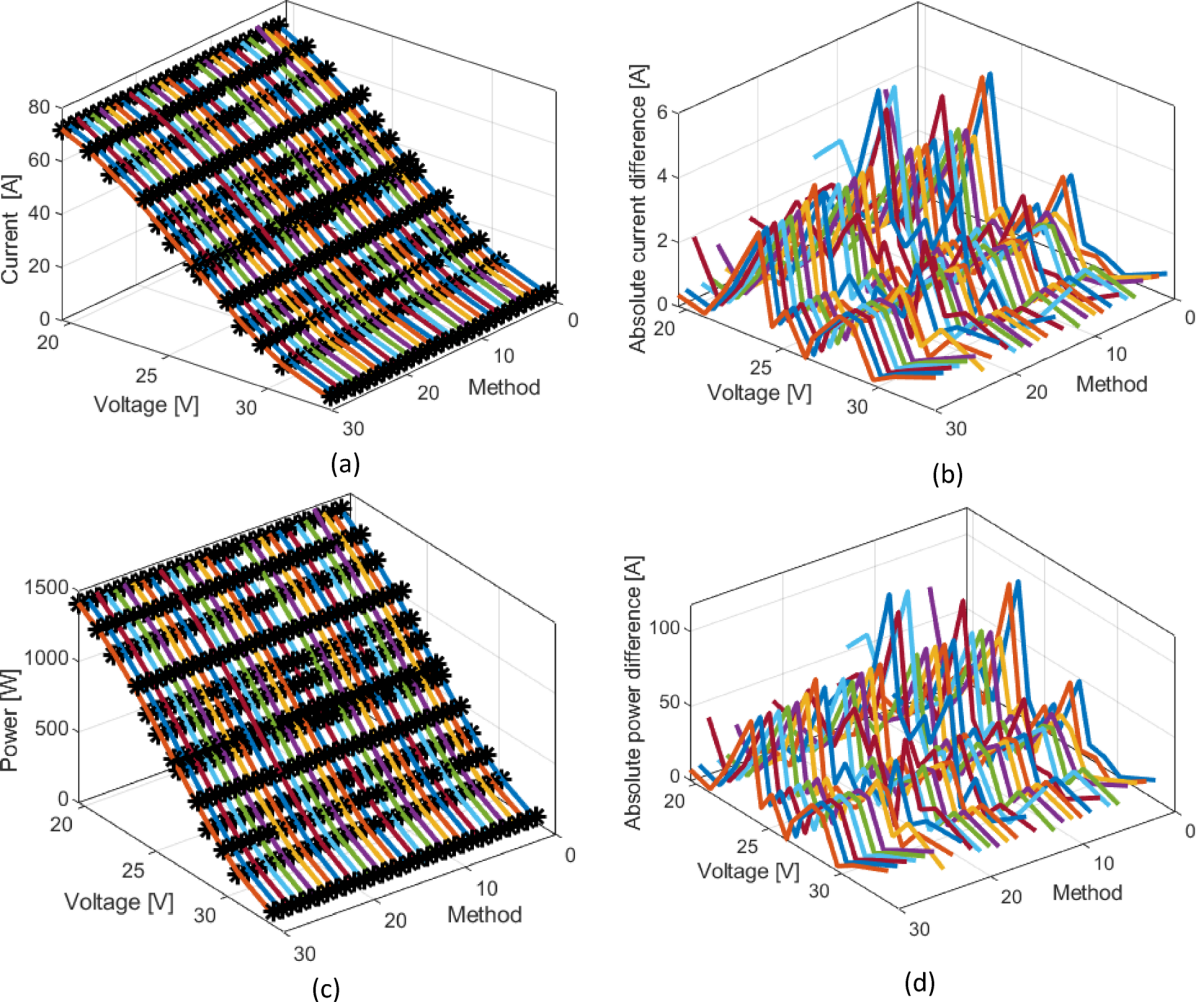
BALLARD PEMFC: (**a**) Current-voltage-method, (**b**) current error-voltage-method, (**c**) Power-voltage-method, and (**d**) Power error–voltage–method curves.

Figure 5a presents a comparison of the obtained current-voltage characteristics using all methods from Table 2 with the measured values for the Ballard fuel cell. Figure 5b shows the corresponding absolute values of current differences, providing a more precise assessment of the deviations between different methods and experimental data. Figure 5c illustrates the power-voltage characteristics, while Fig. 5d shows the absolute power differences, offering additional insight into the model’s accuracy in predicting the fuel cell’s output performance.

As with the RMSE graph, the methods with the highest RMSE values (15, 16, 13, 21, and 14 methods) are noticeable here as well, while many other approaches from the literature provide very similar results.

#### Convergence curves

**Fig. 6:**
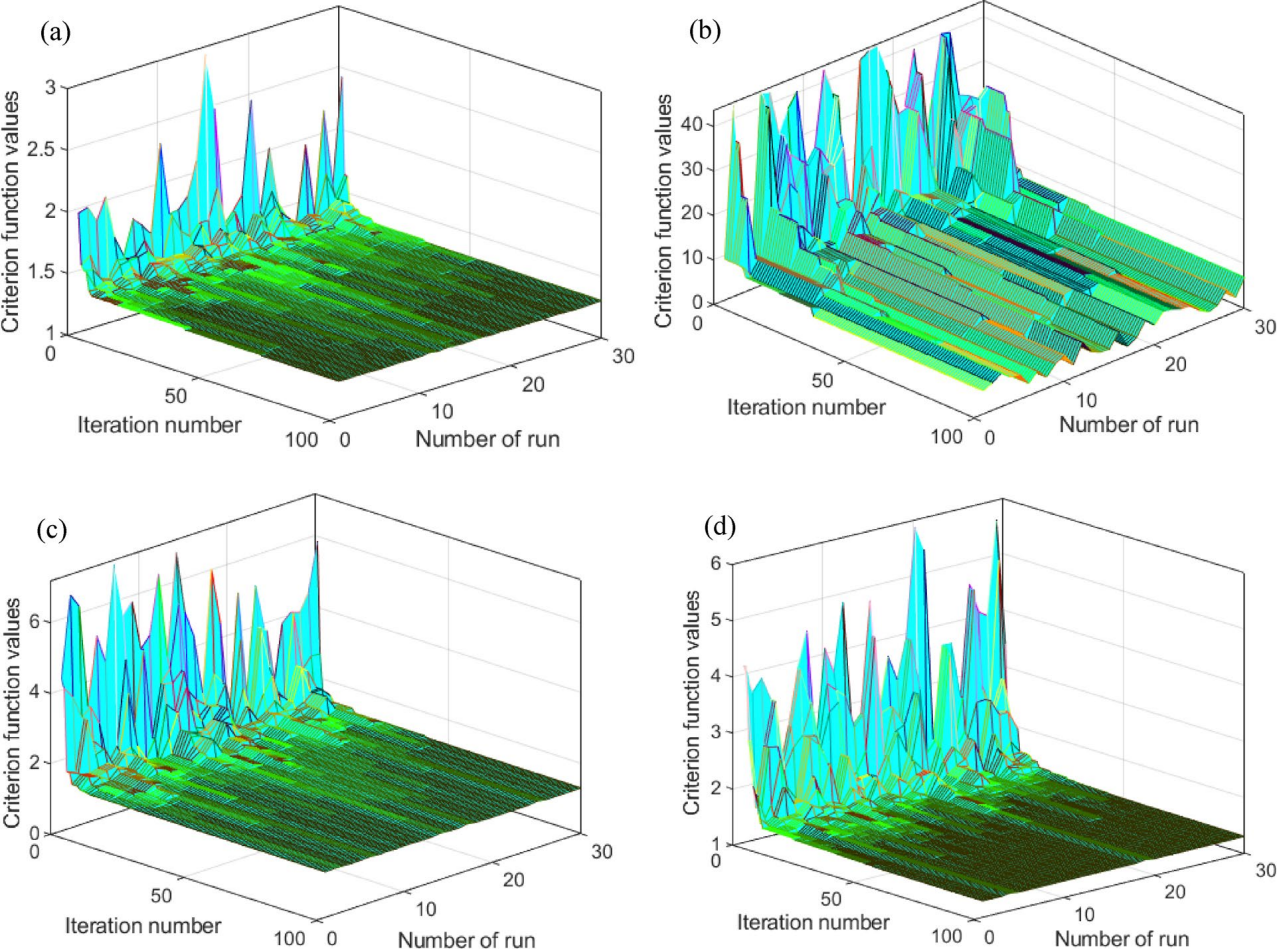
3D convergence curves: (**a**) BFO, (**b**) AVOA, (**c**) POA, and (**d**) SaDE.

**Fig. 7:**
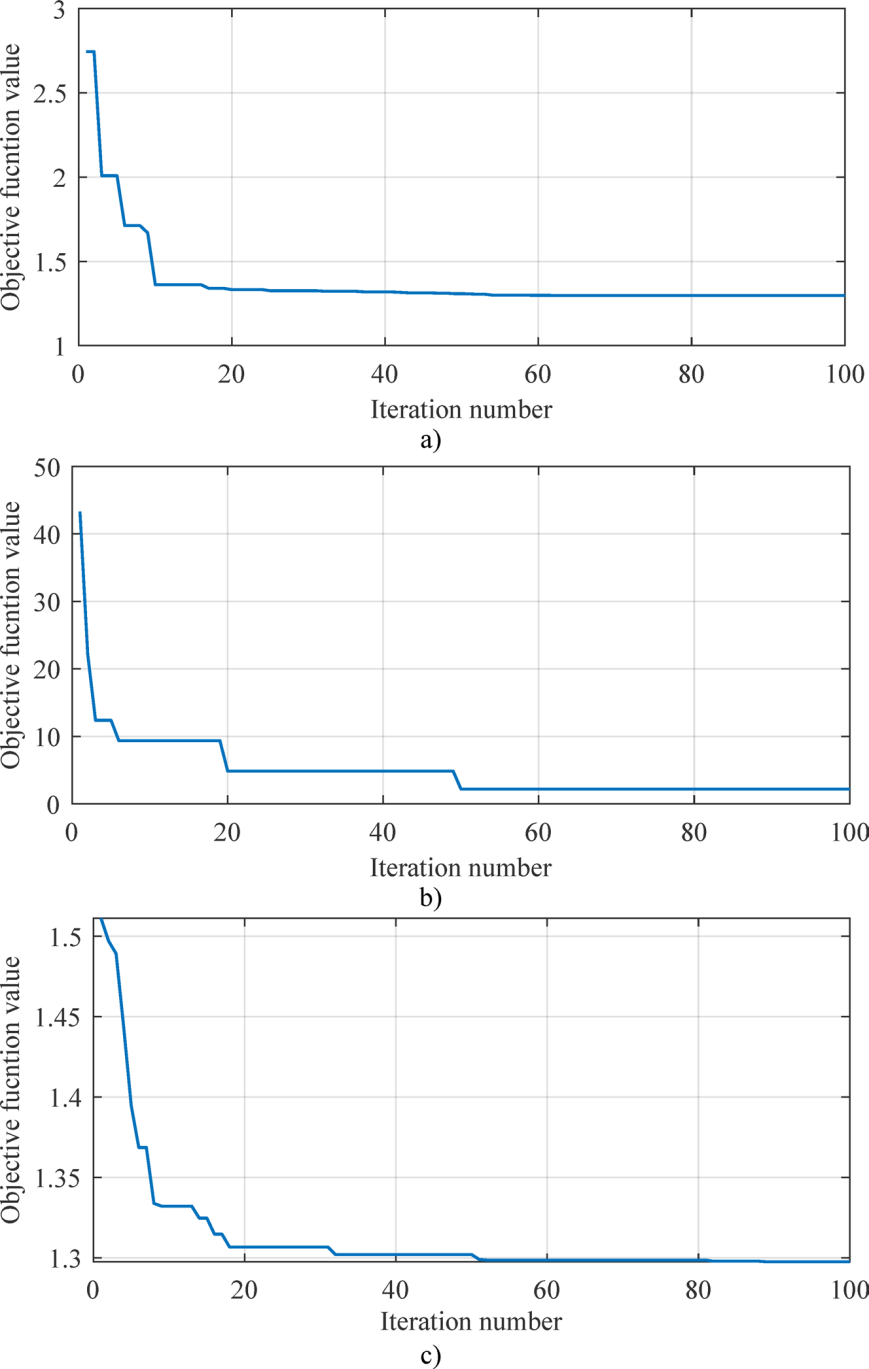

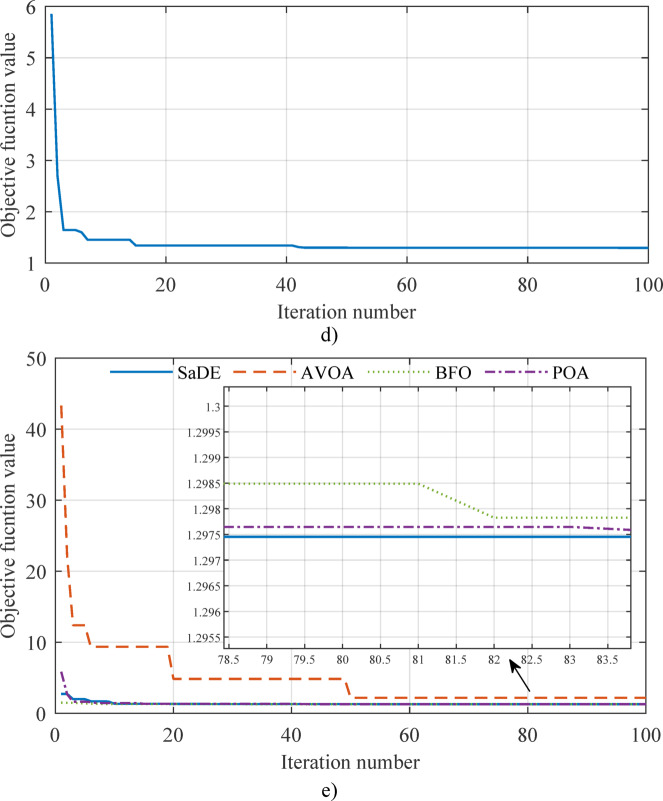
2D convergence curves: (**a**) SaDE, (**b**) AVOA, (**c**) BFO, (**d**) POA, and (**e**) mutual comparison.

In this subsection, a comparison of convergence curves and statistical parameters of several metaheuristic algorithms was conducted, too. In this specific case, in addition to the SaDE algorithm, the African vultures’ optimization algorithm (AVOA)^55^, Pelican optimization algorithm (POA)^56^ and bacterial foraging optimization (BFO)^57^ algorithms were also analyzed. The convergence curves of all four algorithms are shown in Figs. 6 and 7 (3D and 2D comparisons), while the summary results of the statistical parameters (best, worst, mean, median, and standard deviation – STD) are presented in Table 3. It is clearly observable that the proposed SaDE algorithm outperforms all other tested algorithms, which were evaluated under the same conditions—30 test runs, the same objective function (RMSE), and the same computational environment. Apart from the SaDE algorithm, the POA algorithm also yields competitive results in terms of the best outcome; however, regarding all other parameters, particularly STD, it is significantly inferior to the proposed SaDE algorithm. Therefore, the algorithm used in this work is proven to be more applicable compared with the other three popular metaheuristic algorithms.

**Table 3:** Statistical results.

Algorithm	Best	Worst	Mean	Median	STD
BFO	1.297471331363799	1.306989218242300	1.300385643274544	1.299512522056919	0.002842031780050
AVOA	2.194072056634361	7.709276117281034	4.875418307628987	5.126481195274089	1.608367329515606
POA	1.297439246225256	1.342642529019309	1.308054208140956	1.304708517964520	0.012041565099029
SaDE	1.297438280969956	1.320358779269861	1.298944570177995	1.297449725620649	0.005571330141057

#### Statistical tests

Furthermore, various statistical tests have been conducted to examine the statistical significance of the proposed algorithm.

To verify whether the observed performance differences among the algorithms are statistically meaningful, a one-way ANOVA test was conducted over 30 independent runs for each algorithm. The resulting p-value was 1.68 × 10^− 39^, which is far below the 0.05 significance threshold. This indicates that the mean objective values of the compared algorithms differ significantly, confirming that the performance improvements are statistically significant rather than due to random variations.

Additionally, to validate the statistical significance of performance differences among the compared algorithms, pairwise *t*-tests were performed over 30 independent runs. The results show that most algorithm pairs exhibit statistically significant differences (*p* < 0.05), confirming that their performance variations are not due to random effects. Specifically, significant differences were observed between SaDE–AVOA, SaDE–POA, AVOA–BFO, AVOA–POA, and BFO–POA, while the comparison between SaDE and BFO yielded a *p*-value of 0.2119, indicating no statistically significant difference between these two algorithms. These findings further support the robustness of the proposed algorithm’s performance improvements. The exact values of the *t*-test analyses are presented in Table 4.

**Table 4:** p-value from the *t*-tests.

Algorithms	*p*-value
SaDE vs. AVOA	1.2699e-17
SaDE vs. BFO	2.1199e-01
SaDE vs. POA	3.9649e-04
AVOA vs. BFO	1.2913e-17
AVOA vs. POA	1.4146e-17
BFO vs. POA	1.2444e-03

To further confirm the reliability of the statistical analysis, the non-parametric Wilcoxon signed-rank test was applied to all pairwise algorithm comparisons. The obtained *p*-values were below 0.05 in all cases, with *h* = 1 for every pair, indicating that the performance differences are statistically significant across all algorithms. The results obtained with the Wilcoxon test are given in Table 5.

**Table 5:** *p*-value from the Wilcoxon test.

Algorithms	*p*-value
SaDE vs. AVOA	1.7344e-06
SaDE vs. BFO	5.7064e-04
SaDE vs. POA	1.4773e-04
AVOA vs. BFO	1.7344e-06
AVOA vs. POA	1.7344e-06
BFO vs. POA	5.3197e-03

When compared with the results of the parametric *t*-test, the Wilcoxon test provides consistent conclusions, reinforcing that the observed performance improvements are robust and not due to random variations.

To provide a comprehensive ranking of the evaluated algorithms, the Technique for Order Preference by Similarity to Ideal Solution (TOPSIS) method was applied as part of a multi-criteria decision-making (MCDM) framework. Three performance indicators were used as evaluation criteria: the mean value, median value, and the best (minimum) objective function value obtained from 30 independent runs of each algorithm. Since all three criteria represent minimization objectives, smaller values indicate better performance. The relative importance of each criterion was defined by the weight vector w = [0.25, 0.25, 0.50], assigning the highest significance to the best achieved result, while the mean and median values were given equal weight.

The TOPSIS method determines the relative closeness of each algorithm to the ideal best and ideal worst solutions. The resulting scores and rankings showed that SaDE achieved the highest TOPSIS score (1.000), indicating that it is the closest to the ideal solution and therefore provides the most balanced performance across all evaluation criteria. BFO algorithm and POA followed closely with scores of 0.9996 and 0.9981, respectively, demonstrating competitive but slightly inferior results. Finally, AVOA obtained the lowest score (0.000), confirming its relatively weaker performance. A complete overview of the obtained scores and rankings for each algorithm is depicted in Table 6.

**Table 6:** The obtained scores and rankings of different algorithms from the TOPSIS method.

Algorithm	TOPSIS score	Rank
SaDE	1	1
BFO	0.999589826096664	2
POA	0.998081291774992	3
AVOA	0	4

These findings are consistent with the results of the statistical tests, reinforcing that the SaDE algorithm exhibits superior overall performance in terms of accuracy, stability, and reliability across multiple evaluation dimensions.

#### Discussion on the cost-effectiveness of the methods used, addressing computational complexity

The computational cost of the proposed Self-Adaptive Differential Evolution (SaDE) algorithm can be analyzed by decomposing the optimization process into several main stages: population initialization, mutation and crossover operations, evaluation of the objective function, selection and replacement, and periodic adaptation of control parameters and strategies. Each of these stages contributes to the overall computational complexity.

##### Population initialization

At the beginning of the algorithm, an initial population of *NP* individuals, each represented by a *n*-dimensional parameter vector, is generated uniformly within the predefined search boundaries. The cost of this step is linear in both the population size and the problem dimension:

21 $${T_{init}}=O\left( {NP \cdot n} \right).$$

##### Mutation and crossover operations

During each generation, every individual undergoes mutation and crossover to produce a trial vector. These operations involve linear arithmetic combinations of *n*-dimensional vectors. Since both mutation and crossover are applied to all individuals, their combined complexity per generation is:

22 $${T_{mut,cross}}=O\left( {NP \cdot n} \right).$$

This term is relatively small compared to the evaluation cost of the objective function but still grows linearly with the population size and the dimensionality of the problem.

##### Objective function evaluation

This step represents the most computationally demanding part of the algorithm.

For each individual, the objective function (RMSE) is evaluated using *M* measured voltage–current (*V*–*I*) points. For every such point, the corresponding current is computed by solving the PEMFC model based on the *g*-function. The *g*-function must be solved iteratively, requiring on average *k* iterations per point, each involving logarithmic and exponential operations with cost *c*_*g*_​.

Therefore, the computational effort for all individuals in the population during one generation is:

23 $${T_{eval}}=O\left( {NP \cdot M \cdot k \cdot {c_g}} \right).$$

This term usually dominates the total runtime, as each generation requires the full re-evaluation of the population.

##### Selection and replacement

After the objective values are computed, a selection mechanism decides whether each trial vector replaces its parent. This involves a single comparison per individual:

24 $${T_{select}}=O\left( {NP} \right).$$

Additionally, boundary checks and counter updates for successful and unsuccessful strategies are performed, but their computational cost remains linear in *NP*.

##### Adaptive parameter and strategy update

At the end of each learning period of length *L*, the algorithm updates the probabilities of applying different mutation strategies and the statistical parameters related to *F* and *CR*. The adaptation process requires scanning all individuals once to compute success ratios:

25 $${T_{adapt}}=O\left( {\frac{{{G_{\hbox{max} }}}}{L} \cdot NP} \right).$$

##### Total computational complexity

Combining all the components yields the following total cost:

26 $${T_{total}}={T_{init}}+{G_{\hbox{max} }} \cdot \left( {{T_{mut,cross}}+{T_{eval}}+{T_{select}}} \right)+{T_{adapt}},$$

27 $${T_{total}}=O\left( {NP \cdot n+{G_{\hbox{max} }} \cdot NP \cdot \left( {n+M \cdot k \cdot {c_g}} \right)+\frac{{{G_{\hbox{max} }}}}{L} \cdot NP} \right).$$

Since the model evaluation dominates all other operations, the total computational complexity can be approximated as:

28 $${T_{total}}=O\left( {{G_{\hbox{max} }} \cdot NP \cdot M \cdot k \cdot {c_g}} \right).$$

This expression clearly indicates that the overall cost grows linearly with the number of generations, population size, number of measurement points, and average number of internal iterations required by the *g*-function solver.

Although the evaluation step is computationally expensive due to the iterative *g*-function solver, the SaDE algorithm maintains good overall efficiency because of its self-adaptive nature. Unlike the classical Differential Evolution, SaDE automatically tunes the mutation factor *F*, crossover probability *CR*, and strategy probabilities, eliminating the need for exhaustive parameter tuning and multiple restarts. This adaptive behavior substantially reduces the total computational overhead while preserving high estimation accuracy.

Moreover, SaDE achieves a favorable balance between computational cost and performance, as demonstrated by its faster convergence and lower RMSE and standard deviation compared to other metaheuristic algorithms such as BFO, AVOA, and POA. Since the evaluations of individual solutions are independent, the algorithm can also be efficiently parallelized across multiple processors or cores, further improving practical cost-effectiveness without altering its theoretical complexity.

In summary, the proposed SaDE-based parameter estimation method exhibits linear computational scaling with respect to population size, number of generations, and measurement points, while delivering superior accuracy per computational unit, making it both computationally efficient and cost-effective for PEMFC modeling applications.

### Ned stack PS6 6kW PEMFC

**Fig. 8:**
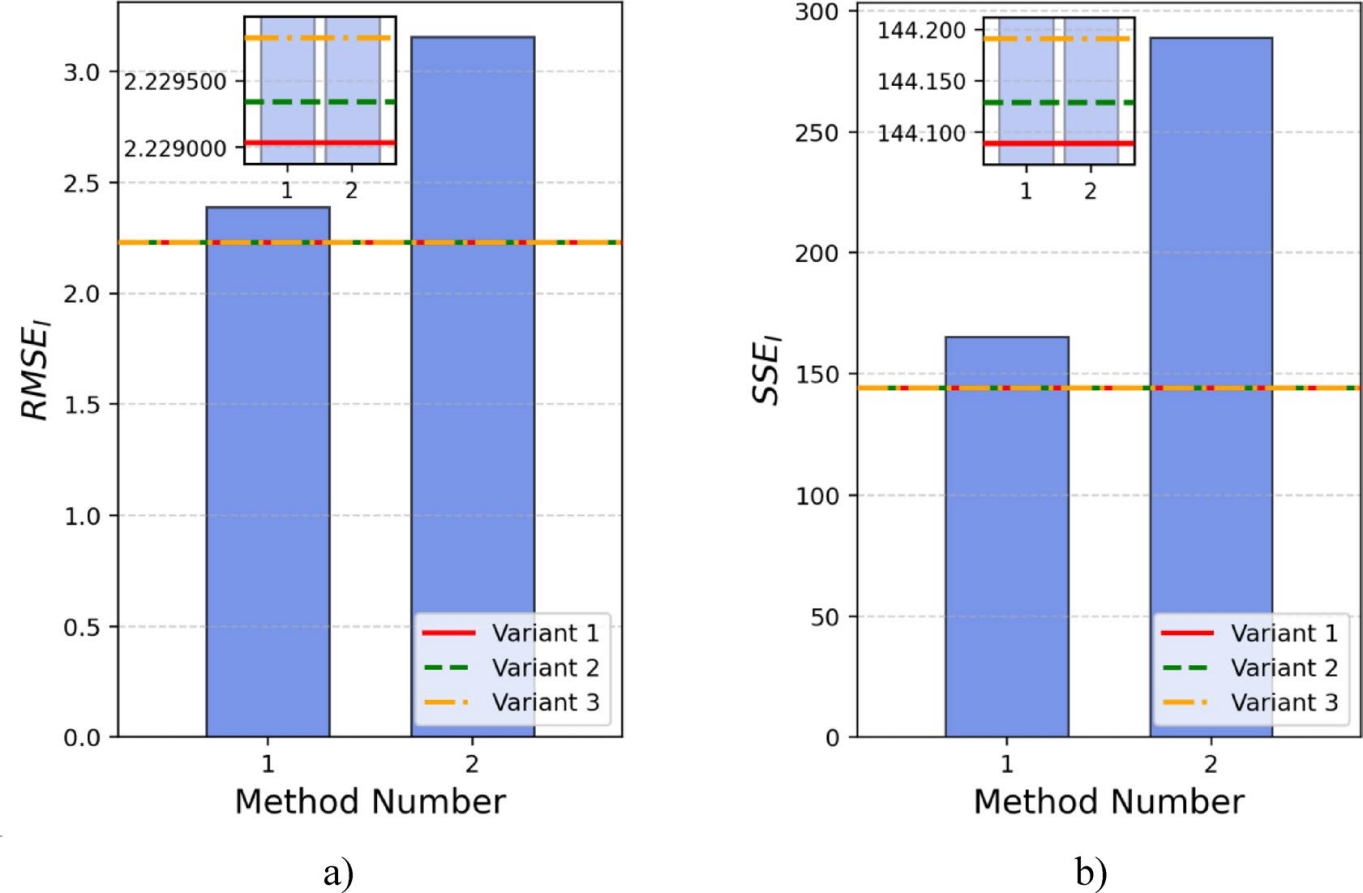
Ned Stack PS6 6 kW PEMFC: (**a**) RMSE and (**b**) SSE comparisons.

Table 7 presents the results from the latest literature for few different methods, along with the results obtained using the SaDE algorithm and the proposed calculation method for the Ned Stack PS6 6 kW PEMFC. Also, the corresponding RMSE_I_ and SSE_I_ values are given. The comparison of RMSE and SSE for this PEMFC is presented in Fig. 8.

For this PEMF, through the optimization process, various values of cell resistance *R*_c_ were explored. Namely, we observed the following variants:

- Variant 1 –The reference configuration is defined with *R*_c_ = 0.1 mΩ. This value was selected as the initial parameter for the optimization process.
- Variant 2 – This configuration features a slightly reduced cell resistance of *R*_c_ = 0.095 mΩ, implemented to analyze its impact on the accuracy of the model.
- Variant 3 – Further reduction of cell resistance**-**The cell resistance is further reduced to *R*_c_ = 0.09 mΩ to examine whether this additional decrease contributes to improving the accuracy of the model.

**Table 7:** Values of parameters obtained by applying the mentioned literature approaches – Ned stack fuel cell.

No.	1	2	3	4	5
Reference	^33^	^37^	SaDE - Variant 1	SaDE - Variant 2	SaDE - Variant 3
Algorithms	MS-TSO	WSO			
*ξ*_*1*_ (V)	-0.8532	-0.85	-1.1477210946	-1.1562935969	-0.8732998898
*ξ*_*2*_ ·10^− 3^ (V/K)	2.5834	2.4	3.5836963389	3.3891246676	2.8727065439
ζ3 · 10^− 5^ (V/K)	4.9206	3.6	5.8809317325	4.3110945774	6.5146362157
ζ4 · 10^− 5^ (V/K)	-9.54	-9.54	-9.54	-9.54	-9.54
λ	12.5734	12.57	12.3469862895606	12.3420334275	12.3853559878
Rc(mΩ)	0.1	0.1	0.09	0.095	0.1
*β* (V)	0.0136	0.01	0.0195957408	0.0136	0.0136
RMSE_I_(A)	2.3879707523	3.1565682538	2.22903624419959	2.22934484992504	2.2298238063967
SSEE_I_ (A²)	165.3697251034	288.9537710789	144.0894747607	144.1293753367	144.1913120196

Based on the data in Table 7, it is evident that the results obtained using the MS-TSO algorithm (No. 1 in the mentioned table), with an RMSE_I_ of 2.3879707523 and an SSE_I_ of 165.3697251034, as well as those obtained with the WSO algorithm, are inferior to the outcomes achieved by the proposed methods. All three proposed variants consistently outperform the literature approaches, confirming the stability and effectiveness of the developed methodology. Among them, Variant 1 delivered the best performance, with an RMSE_I_ of 2.22903624419959 and an SSE_I_ of 144.0894747607.

**Fig. 9:**
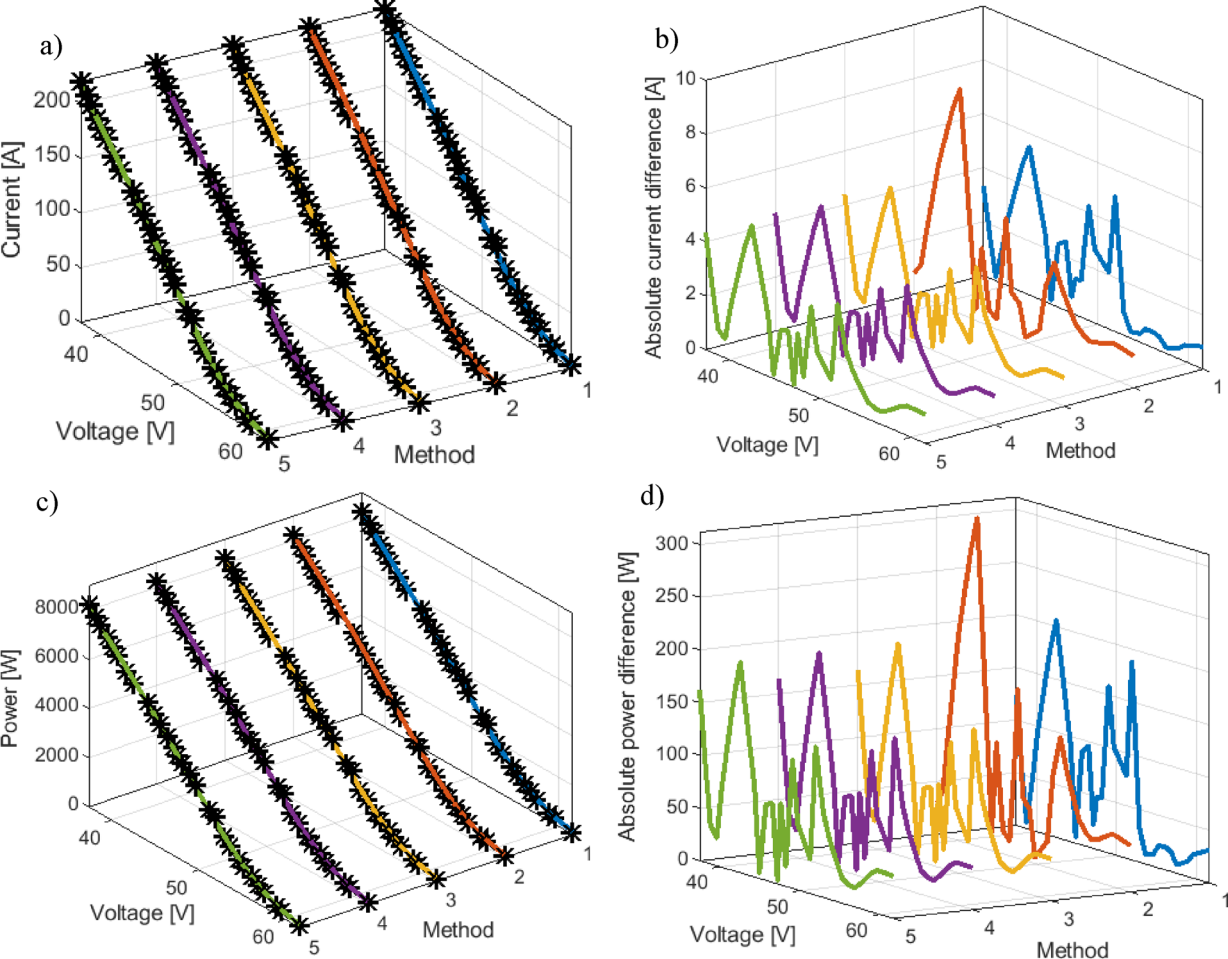
NedStack PEMFC: (**a**) Current-voltage-method, (**b**) current error-voltage-method, (**c**) Power-voltage-method and (**d**) Power error-voltage –method curves.

To further examine the accuracy of the proposed methodology, Fig. 9a compares the current–voltage characteristics of all methods from Table 7 with the experimental data for the NedStack PS6 PEMFC, enabling a direct evaluation of their agreement with measured behavior. Figure 9b complements this by showing the absolute current differences, offering a clearer picture of the discrepancies between simulated and experimental results. In addition, Fig. 9c presents the power–voltage characteristics, while Fig. 9d depicts the absolute power differences. Altogether, these comparisons provide a detailed assessment of how effectively the proposed model predicts the real operating performance of the NedStack PS6 system.

### BSC PEMFC

**Fig. 10:**
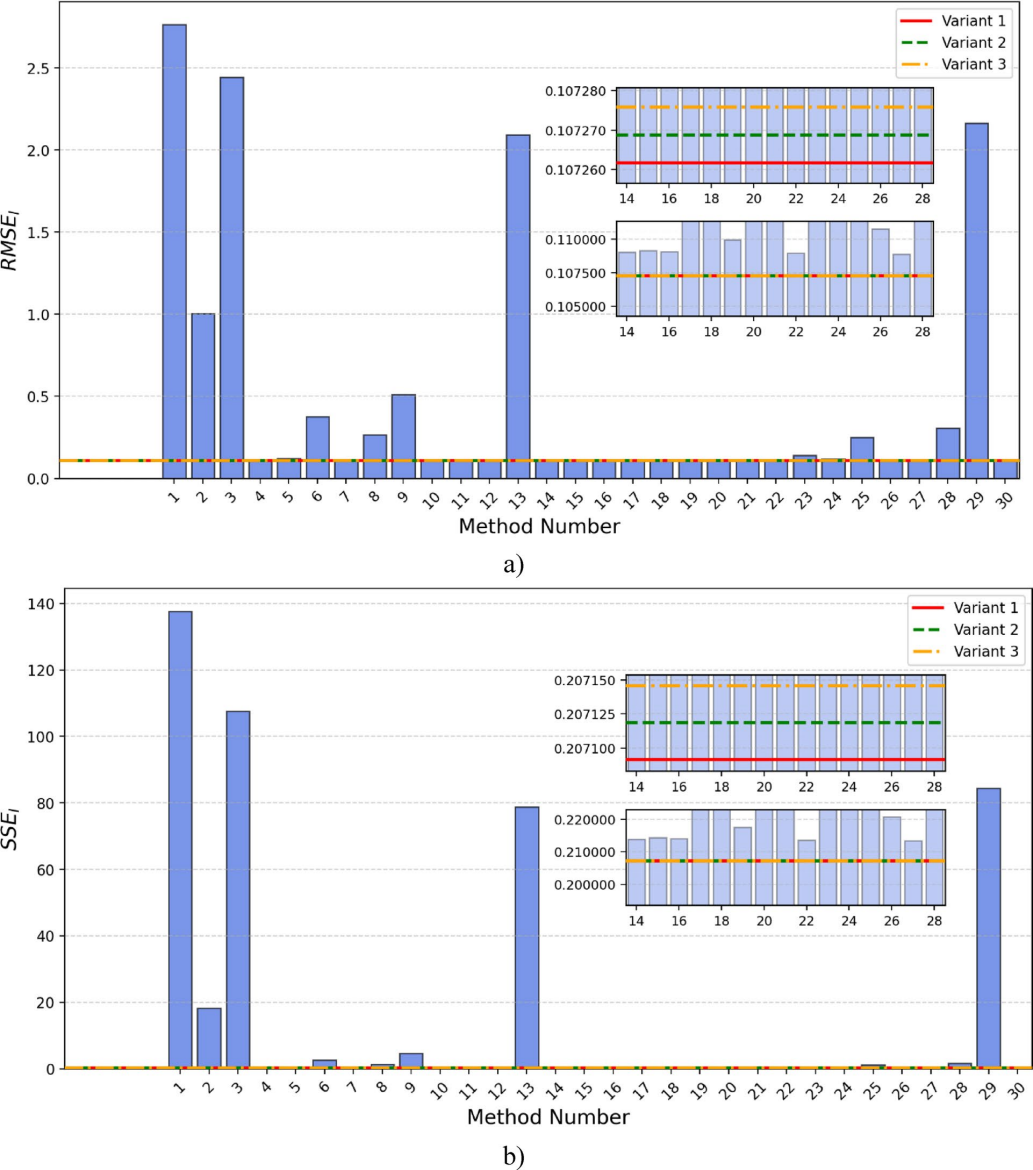
BSC PEMFC: (**a**) RMSE and (**b**) SSE comparisons.

Table 8 presents the results from the latest literature for 30 different methods, along with the results obtained using the SaDE algorithm and the proposed calculation method for the BCS 500 fuel cell. Additionally, the corresponding RMSE_I_ and SSE_I_ values are provided. The comparison of RMSE and SSE for this PEMFC is presented in Fig. 10.

In this case, the optimization process revealed the same variance as observed for the NedStack PS6 6 kW PEMFC (as explained in the previous section).

**Table 8:** Values of parameters obtained by applying the mentioned literature approaches - BCS 500 W stack fuel cell.

*N*	L	Algorithms	Journal	ξ_1_ (V)	ξ_2_ ·10 − 3 (V/K)	ζ3 · 10 − 5 (V/K)	ζ4 · 10 − 5 (V/K)	λ	Rc(mΩ)	β (V)	RMSE_I_(A)	SSEE_I_ (A^2^)
1	^26^	ESSA	Int. J. Hydrogen Ener.	−0.8532	2.2577	3.60000	−19.275	20.7722	0.10007	0.016249	2.7657725097	137.6909563574
2	^27^	WNTGWO	Int. J. Hydrogen Ener.	-0.8532	2.8105	8.0883	-12.887	14.3197	0.16833	0.033890	1.0058620304	18.2116516356
3	^28^	MSMA	Int. J. Hydrogen Ener.	-1.1996	3.14126	3.60029	-19.265	22.0849	0.213978	0.01261	2.4441648335	107.5309511997
4	^29^	AEA	Int. J. Hydrogen Ener.	-0.8794	2.336	4.11	-19.3	20.8755	0.1	0.0161	0.1147340237	0.2369501315
5	^30^	MNEARO	Scient. Reports	-0.9259692	2.6293	5.08	-19.3	20.877243	0.1	0.016261	0.1213687781	0.2651468454
6	^7^	DTBA	Scient. Reports	-0.8581	2.3739	4.7597	-19.2863	21.9631	0.0203	0.0162	0.3767967727	2.5555645425
7	^6^	KOA	Scient. Reports	-1.1654	3.9388	8.9029	-19.2956	20.9077	0.1	0.0161	0.1093272089	0.2151438949
8	^6^	PSA	Scient. Reports	-0.9006	2.5922	5.3451	-19.2701	21.0882	0.0136	0.0161	0.2666257899	1.2796076131
9	^8^	EBOA	PeerJ. Comput. Sci.	-0.9966	2.8534	5.17	-19.3518	22.8929	0.0016	0.0161	0.5094619830	4.6719272182
10	^8^	ARA	Sustainability	-1.1762	3.7344	7.3729	-19.3017	20.8772	0.1	0.0161	0.1090341410	0.2139919903
11	^31^	FFA	Energy	-0.9928	2.621	3.7464	-19.3	21.1011	0.1	0.0161	0.1164088624	0.2439184184
12	^16^	OOA-COA	Energy	-1.14908	2.983130	3.05394635	-19.30303	20.776096	0.09	0.016120	0.1088281528	0.2131842032
13	^17^	Nema skr	Energy	-1.1042	3.3160	6.0640	− 19.301	13.4649	0.10894	0.0161	2.0918070681	78.7618225828
14	^13^	ERWCA	Energy	-1.1742	3.1597	3.7063	-19.3017	20.8772	0.1	0.0161	0.1090231044	0.2139486713
15	^14^	ABCDEA	Energy	-1.1706	4.0932	9.7961	-19.3017	20.8772	0.1	0.0161	0.1091276239	0.2143590894
16	^32^	JSA	Energy	-0.9689	2.693	4.67	-19.3	20.8389	0.1	0.0161	0.1090576887	0.2140844304
17	^31^	SFLA	Energy	-0.96574	3.08	7.2236	-19.3	20.88622	0.1	0.01616	0.1116713471	0.2244688157
18	^31^	ICA	Energy	-0.908643	2.4798	4.4583194	-19.3	22.66264	0.246	0.016238	0.1150956729	0.2384462506
19	^31^	FOA	Energy	-0.992829	2.621	3.74636	-19.3	21.101126	0.1	0.016269	0.1099311049	0.2175272608
20	^33^	MS-TSO	Energies	-1.0214	3.4385	8.4555	-19.302	20.8772	0.1	0.0161	0.1160053784	0.2422304607
21	^18^	LSA	Energies	-1.0134	2.9662	5.5693	-19.2904	20.93	0.1	0.0161	0.1154040732	0.2397258020
22	^24^	CSA	Electron	-1.17659134	3.496528	5.8319	-19.2897	21.32420587	0.146406	0.01614053	0.1089351642	0.2136036600
23	^24^	NNA	Electron	-0.87290963	3.189571	9.7398	-18.5976	22.99629125	0.8	0.01363004	0.1385485710	0.3455227175
24	^24^	GWO	Electron	-1.19520326	3.627031	6.3151	-19.12	19.26220378	0.116867	0.01490063	0.1190019661	0.2549064228
25	^24^	SCA	Electron	-0.86756578	2.527557	5.3856	-20.1822	23	0.164497	0.01891043	0.2489101737	1.1152129423
26	^34^	GWO-CS	Int. J. Energy Res	-1.04401	3.251	6.82	-19.118	22.48551	0.362	0.015624	0.1107684595	0.2208537292
27	^34^	CMOA	Int. J. Energy Res	-0.85321	3.141	9.8	-19.2993	20.86005	0.1	0.016117	0.1088899097	0.2134262238
28	^35^	EAHA	Int. J. Energy Res.	-0.95359	2.823	5.8	-19	22.89328	0.32	0.015792	0.3069821519	1.6962847485
29	^36^	INFONIM	IEEE ACCESS	-1.1230	3.4501	6.5693	-19.481	13.822	0.17736	0.015326	2.1652812873	84.3919749564
30	^25^	L-AHA	Measurement	-0.9040	3.0511	8.2338	-19.285	20.7067	0.1	0.015958	0.1120884011	0.2261485739
31	SaDE - Variant 1			-1.1642001797	3.647753345062	7.07902941034	-18.9408628585	19.66794030167	0.090	0.01584570134661	0.1072617068	0.2070913274
32	SaDE - Variant 2			-1.1724443004	3.739219624080	7.50924856636	-18.9392712290	19.71032086327	0.095	0.01584856632475	0.1072687434	0.2071184996
33	SaDE - Variant 3			-1.0404987886	3.08715226515	5.859645583493	-18.9378376218	19.75336994038	0.100	0.01585162280987	0.1072757771	0.2071456623

The analysis of the results indicates that, among the methods from the literature, the OOA-COA method (method No. 13 in Table 8) achieved the best result, with the lowest RMSE_I_ value of 0.1088281528. In this case, as well, all three variants implemented using the SaDE algorithm and the proposed calculation model exhibited lower error values compared to all methods from the literature. The best result was obtained using Variant 1, which had an RMSE_I_ value of 0.10726 and SSE_I_ of 0.20709.

This represents an improvement compared to the best method from the literature (OOA-COA, RMSEI = 0.1088281528).

**Fig. 11:**
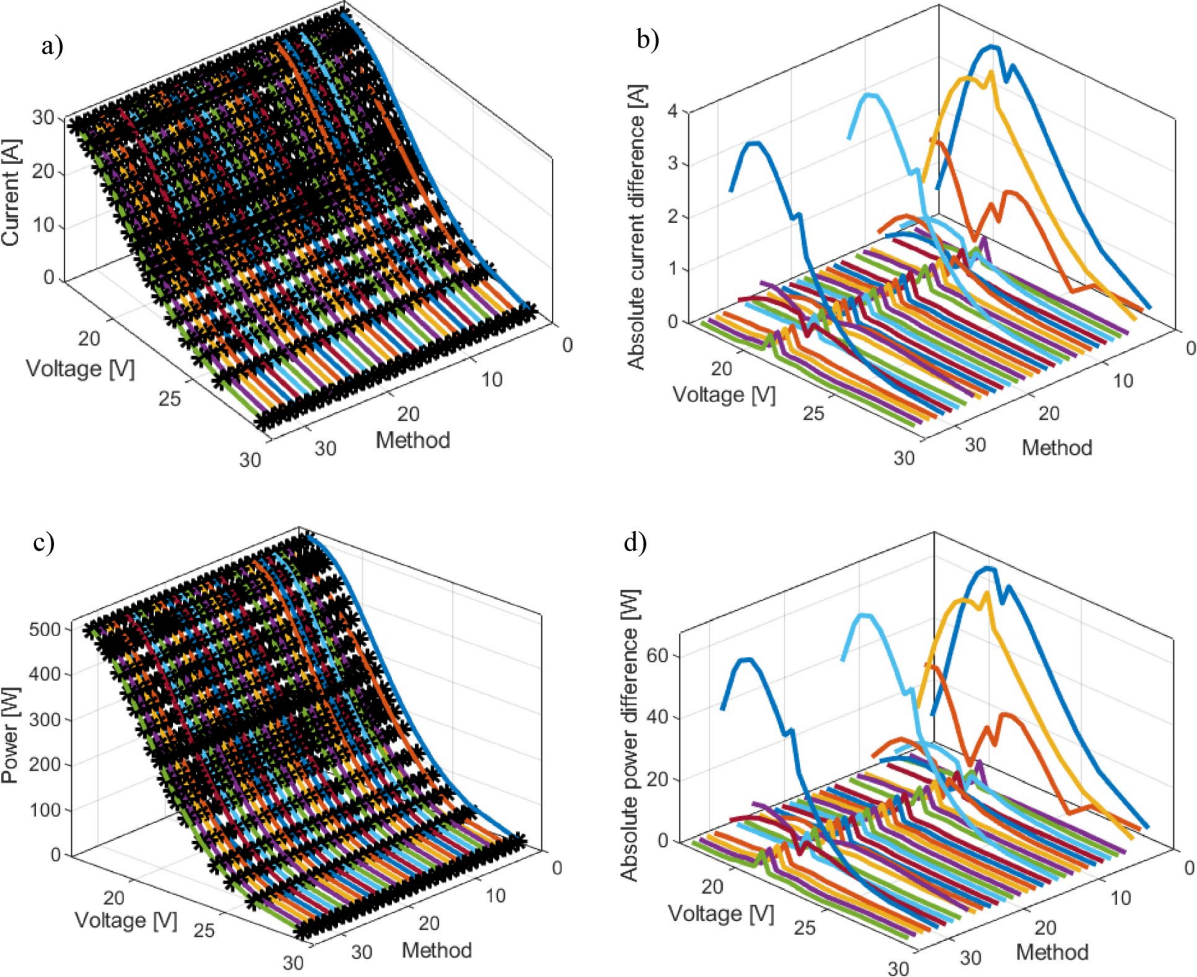
BSC PEMFC: (**a**) Current-voltage-method, (**b**) current error-voltage-method, (**c**) Power-voltage-method and (**d**) Power error-voltage –method curves.

To gain a deeper insight into the model’s accuracy, Fig. 11a presents a comparison of the current-voltage characteristics obtained using all methods from Table 8 with experimental data for the BCS 500 W fuel cell. This graphical representation enables a visual assessment of the precision of individual models relative to actual values. Additionally, Fig. 11b illustrates the absolute values of current deviations, providing a more detailed analysis of the differences between the simulated and measured data.

To evaluate and assess the fuel cell’s output performance in terms of power, Fig. 11c presents the power-voltage characteristics, while Fig. 11d depicts the absolute power differences. This provides additional insight into the model’s accuracy in predicting real operating conditions of the fuel cell.

Figures 10 and 11 indicate that methods 1, 3, 29, 13, and 2 exhibit the highest RMSE values within the analyzed set. A comparable pattern is evident in Fig. 10b, where the SSE results further highlight the increased error levels associated with these methods.

The proposed parameter optimization method, utilizing the SaDE algorithm, has demonstrated superiority over existing methods in the literature. The obtained results suggest that the application of optimized variants can achieve a lower RMSE_I_ value and enable more accurate modeling of the real current-voltage characteristics of PEM fuel cells. On the second side, this analysis demonstrates that reducing the enclosure resistance *R*_c_ ​ enhances the accuracy of current-voltage characteristic modeling for the fuel cell. This improvement enables more precise performance predictions across various operating conditions.

**Fig. 12:**
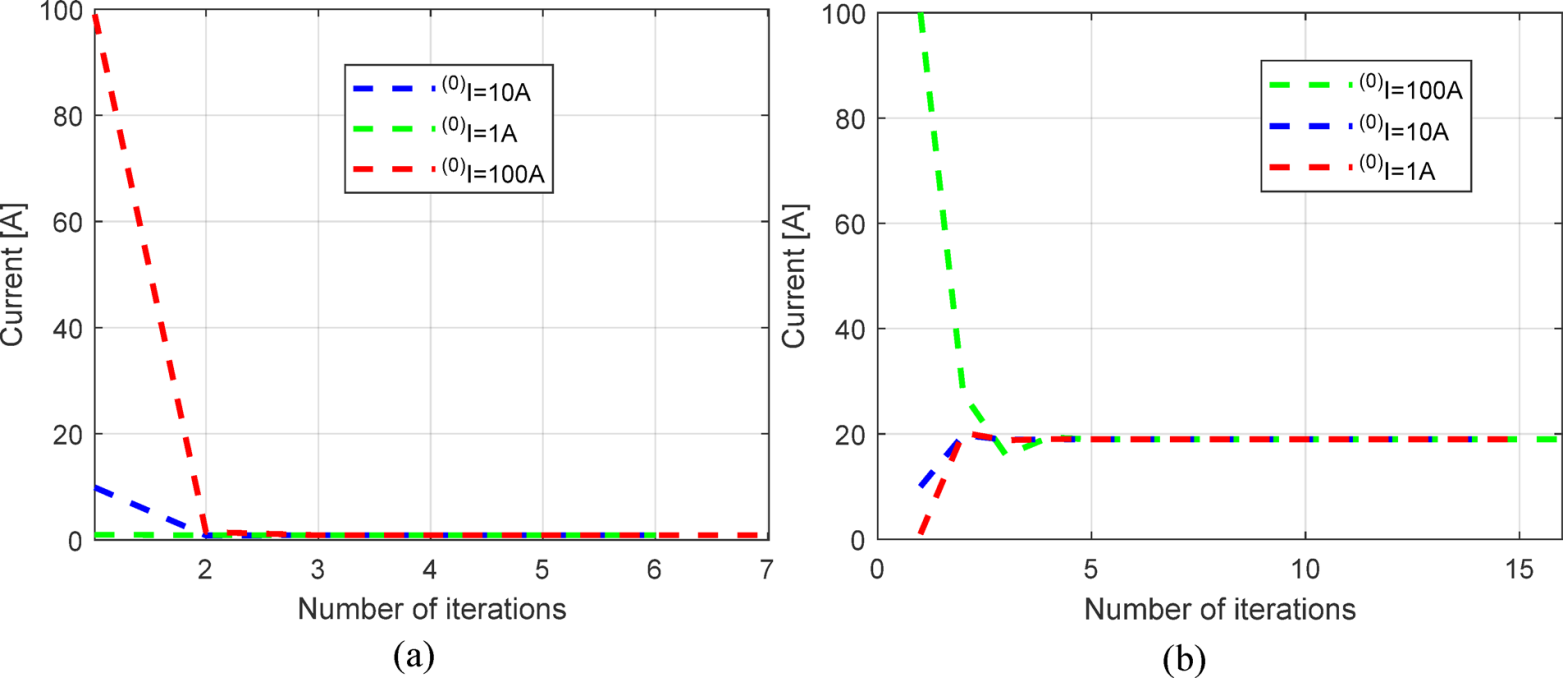
Required number of iterations for: (**a**) voltage of 29.06 V and (**b**) voltage of 21.09 V.

In addition to previous research, this section presents the results of an investigation into the impact of the convergence criterion on the required number of iterations in the proposed iterative procedure (see Fig. 12). To this end, two cases were considered: the first point (voltage of 29.06 V) and the tenth point (voltage of 21.09 V) with a convergence criterion of 10^–15^. The proposed iterative procedure is highly efficient and consistently converges in the same number of iterations, regardless of the initial current value. Furthermore, the procedure requires fewer iterations for higher voltage values. These findings confirm the robustness of the proposed iterative procedure and the high efficiency of the parameter estimation approach.

## Testing the model and the algorithm employed in this work under various operating conditions

This section consists of two parts. The first part analyzes the impact of insolation and temperature on the characteristics of the PEMFC, while the second part tests the sensitivity of the calculated parameters to the output characteristics of the PEMFC.

### The impact of temperature and pressure changes

As is well known, variations in operating conditions, particularly temperature and pressure, have a significant impact on the performance of fuel cells. Therefore, it is crucial to analyze their stability and robustness under variable operational environments. Therefore, in this section, the impact of temperature and pressure changes on the parameter estimation results of the Ballard-Mark-V 5 and BCS 500 fuel cells is analyzed.

**Fig. 13:**
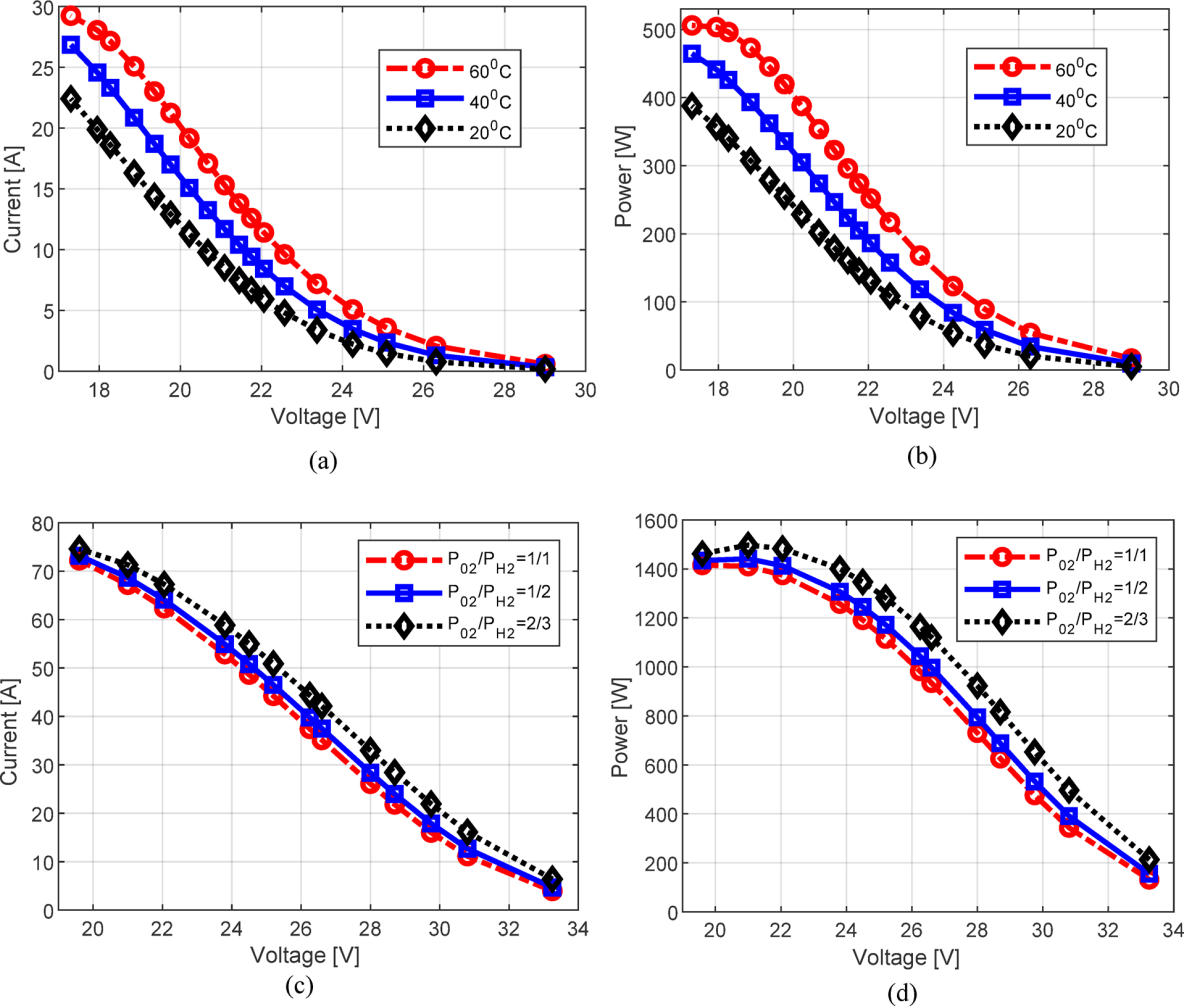
Impact of temperature on the BCS PEMFC: (**a**) current-voltage and (**b**) power-voltage characteristics as well as the impact of pressure on the BALLARD PEMFC on (**c**) current-voltage and (**d**) power-voltage characteristics.

In this section, to ensure clarity and conciseness of the presented results, the effect of temperature variation is analyzed for the BCS fuel cell, while the impact of pressure changes is observed for the Ballard fuel cell. Specifically, temperature values of 20 °C, 40 °C, and 60 °C were considered, along with pressure ratios of 1/1, 1/2, and 2/3. The results obtained are depicted in Fig. 13. From the figure, several key characteristics of PEMFCs can be observed. First, their operation, and consequently their current-voltage characteristics, are strongly influenced by both temperature and pressure values. For example, increasing the temperature from 20 °C to 60 °C leads to a current increase of 8.16 A at a voltage level of 17.95 V. Similarly, variations in pressure also have a pronounced impact, with higher pressure ratios contributing to improved output characteristics. For instance, at a voltage of 22.05 V, increasing the pressure ratio from 1/1 to 2/3 results in a current increase of 4.91 A, clearly highlighting the beneficial effect of elevated pressure on the performance of the fuel cell.

**Fig. 14:**
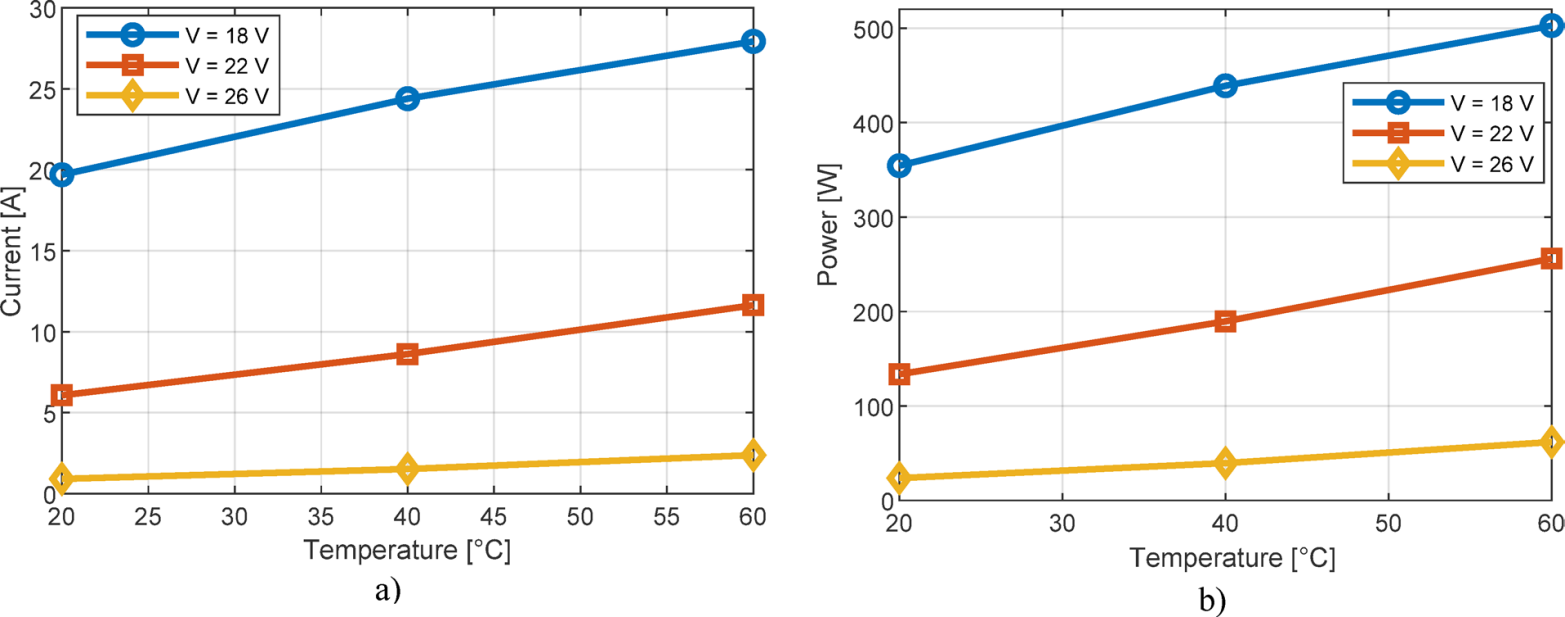
Impact of temperature on PEMFC: (**a**) current, (**b**) power.

**Fig. 15:**
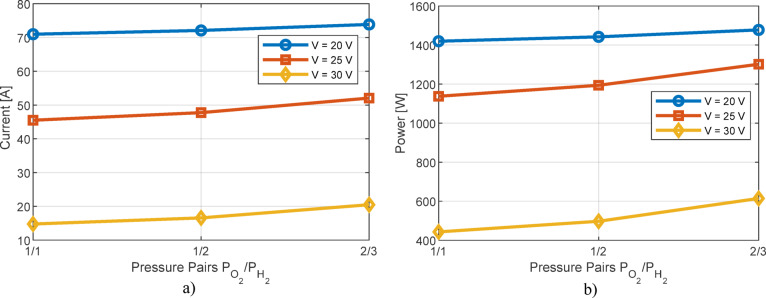
Impact of temperature on PEMFC: (**a**) current, (**b**) power.

Apart from the previous research, the detailed influence of temperature on the current value for various voltage levels at the terminals of the PEMFC is shown in Fig. 14a. The corresponding results for the influence of temperature on power are presented in Fig. 14b. These results apply to the Ballard PEMFC. The influence of pressure on current and power values is shown in Fig. 15. These results are in complete agreement with Fig. 13 and the explanations regarding the influence of temperature and pressure on the output characteristics of PEMFC cells.

### Sensitivity analysis

In this section, a sensitivity analysis was performed to examine in more detail the impact of variations in individual parameters on the estimated output characteristics of the fuel cell and their relationship with the objective function value, specifically the current RMSE metric. To achieve this, a systematic variation of each parameter within a ± 10% range was performed to quantify its effect on the final results. This analysis provides a deeper understanding of the model’s sensitivity and helps identify the key factors that have the most significant influence on its accuracy and predictive capabilities.

To quantify sensitivity, the function *Y* has been defined as follows.

29 $$Y=\frac{1}{N}\sum\limits_{{m=1}}^{M} {|\frac{{\left( {I_{m}^{{meas}} - I_{m}^{{calc}}} \right)}}{{I_{m}^{{meas}}}}|} \times 100$$

allowing for a thorough and precise analysis of how variations in individual parameters affect the value of the objective function. This approach enhances the understanding of their relationships and overall significance within the model.

**Fig. 16:**
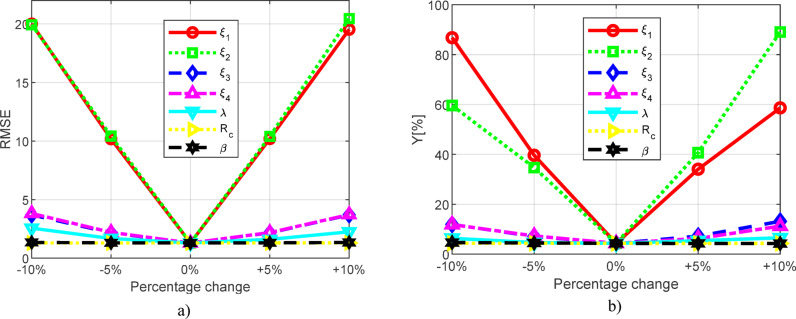
Graphical presentation of sensitivity analyses (Ballard Mark V fuel cell) for: (**a**) RMSE and (**b**) Y.

The results for the Ballard Mark V fuel cell, specifically the RMSE and *Y* values, are presented in Table 9. The obtained results are graphically displayed in Fig. 16. It can be observed that in the proposed model, the most significant influence comes from the factor _2_, where a 10% increase leads to an RMSE value of 20.461142129 and a corresponding *Y* value of 89.051778944%. Similarly, the factor _1_ also has a considerable impact, as a 10% decrease results in an RMSE value of 20.008312784, while *Y* amounts to 86.776654093%. In contrast, the least impact on the RMSE value is observed for the resistance *R*_*c*_, with an error of 1.2972422551 and a corresponding *Y* value of 4.3812733193%, as well as for , which has an error value of 1.3439104938 and a corresponding *Y* of 4.6601593126%.

**Table 9:** Sensitivity analysis for Ballard PEMFC.

Variable	Percentage change (%)	RMSE	Y (%)
Original case	0	1.2963193226	4.3337009740
*ξ*_*1*_	-10%	20.008312784	86.776654093
	-5	10.177731702	39.647193027
	+ 5%	10.194683525	34.056300757
	+ 10%	19.527919147	58.642306403
*ξ*_*2*_	-10%	19.933704687	59.591420143
	-5	10.421690709	34.721423673
	+ 5%	10.404253688	40.666957730
	+ 10%	20.461142129	89.051778944
*ξ*_*3*_	-10%	3.6890828857	11.923845092
	-5%	2.1613333865	7.0914679224
	+ 5%	2.1600160500	7.2292252247
	+ 10%	3.6909513436	13.164898101
*ξ*_*4*_	-10%	3.8188115588	11.857112540
	-5	2.2063985890	7.2978721483
	+ 5%	2.1845145969	6.2883506798
	+ 10%	3.7186963956	11.233395309
λ	-10%	2.5609372588	6.3496006077
	-5	1.6798566798	4.6425640467
	+ 5%	1.6060477988	5.3954134122
	+ 10%	2.2468622115	6.5638039235
R_c_	-10%	1.2972422551	4.3812733193
	-5	1.2963032206	4.3539035248
	+ 5%	1.2972880960	4.3222673790
	+ 10%	1.2992049796	4.3108398049
*β*	-10%	1.3439104938	4.6601593126
	-5	1.3082760819	4.4776058424
	+ 5%	1.3077200250	4.2779812685
	+ 10%	1.3409827263	4.2606551402

**Fig. 17:**
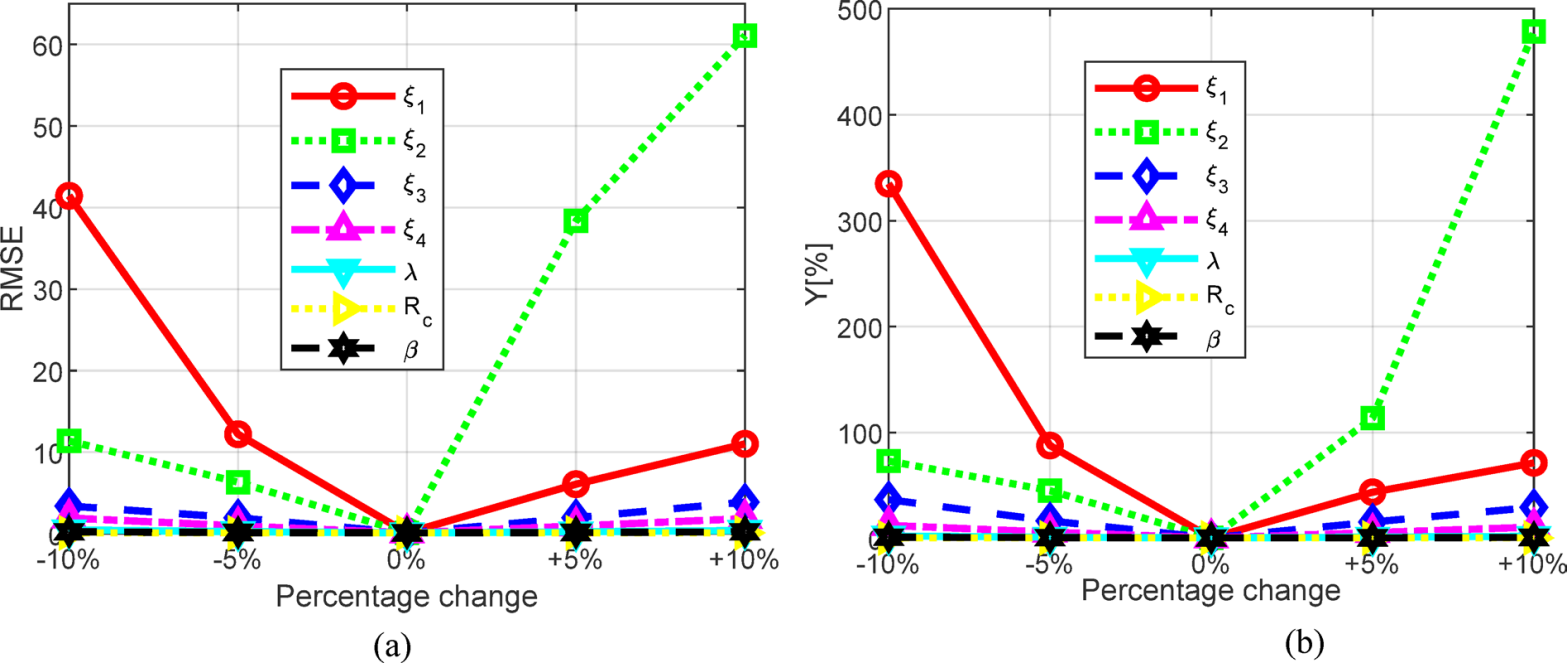
Graphical presentation of sensitivity analyses (BCS 500 W fuel cell for: (**a**) RMSE and (**b**) Y.

The corresponding results for the BCS fuel cell are presented in Table 10, with the graphical representation provided in Fig. 17. Similar to the previous case, it is evident that the most significant influence comes from the change in factor _2_, where a 10% increase leads to an RMSE value of 61.086619987 and a corresponding *Y* value of 478.49006344%. Similarly, factor _1_ also has a considerable impact, as a 10% decrease results in an RMSE value of 41.437572693, while *Y* amounts to 334.91104754. The least impact on the RMSE value is observed for *R*_*c*_, with an error of 0.1087081572 and a corresponding *Y* value of 0.7231442269%, as well as for , which has an error value of 0.2361495887 and a corresponding *Y* of 1.1242757382%.

**Table 10:** Sensitivity analysis for BSC PEMFC.

Variable	Percentage change (%)	RMSE	Y (%)
Original case	**0**	**0.1072617068**	**0.6697478354**
*ξ*_*1*_	-10%	41.437572693	334.91104754
	-5	12.200579934	87.893142798
	+ 5%	6.0754144369	43.808165727
	+ 10%	11.019334677	71.481478222
*ξ*_*2*_	-10%	11.369828418	73.231866307
	-5	6.3207657407	45.323716161
	+ 5%	38.356239329	113.80656531
	+ 10%	61.086619987	478.49006344
*ξ*_*3*_	-10%	3.4002448235	36.822338000
	-5%	1.9515324981	16.741495944
	+ 5%	1.9612498901	15.714890977
	+ 10%	3.8884618059	29.543011946
*ξ*_*4*_	-10%	1.9283485158	12.521109403
	-5	0.9626446962	6.1570195979
	+ 5%	0.9456115598	5.6821400506
	+ 10%	1.8593613622	10.892483926
λ	-10%	0.4771730233	2.5198654417
	-5	0.2452740911	1.3443234005
	+ 5%	0.2268765469	1.3971349026
	+ 10%	0.3966264171	2.1169434009
R_c_	-10%	0.1087081572	0.7231442269
	-5	0.1076218104	0.6923618150
	+ 5%	0.1076342287	0.6566029052
	+ 10%	0.1087309462	0.6507679175
*β*	-10%	0.2361495887	1.3642762904
	-5	0.1496392776	1.0077183415
	+ 5%	0.1484650539	0.7702489409
	+ 10%	0.2301458506	1.1242757382

The proposed model is based on the *g*-function, which overcomes numerical limitations associated with the Lambert W function and improves computational stability. However, its accuracy depends on the assumptions made during its formulation. Real operating conditions of PEMFC cells, such as fluctuations in pressure, temperature, and humidity, can lead to deviations from the modeled characteristics. Since the model primarily describes the static voltage-current relationship, it does not account for dynamic effects, such as transient temperature and pressure variations or changes in load. This limitation may affect its accuracy in applications requiring rapid response, such as electric vehicles and microgrids. While validated using literature data, the model has not been experimentally verified under real operating conditions, which may limit its applicability in industrial and commercial settings. Additionally, the model relies on Mann’s PEMFC framework, which employs approximations to simplify complex processes like mass transport and thermal effects. Although these approximations reduce computational complexity, they can introduce discrepancies between simulated and actual fuel cell performance. To enhance reliability and practical usability, future research should incorporate dynamic system analysis and experimental validation under real operating conditions.

## Conclusion

This paper presents a novel methodology for estimating the output characteristics of PEMFC fuel cells using the self-adaptive differential evolution (SaDE) algorithm. Unlike traditional modeling approaches that rely on voltage-current (*V*-*I*) characteristics, this research introduces an innovative method based on current-voltage (*I*-*V*) characteristics, enhancing the model’s adaptability to real operating conditions and improving its numerical stability.

One of the key contributions of this research is the use of the *g*-function to solve the equation describing the operation of PEMFC fuel cells. This approach overcomes the numerical limitations of the Lambert W function, particularly in cases where its application becomes impractical due to excessively large arguments. By utilizing the *g*-function, the stability and accuracy of calculations are significantly improved, which is crucial for the application of the model in fuel cell simulations and optimization.

The validation of the proposed methodology was conducted on two well-known and promising types of PEMFC fuel cells, which are considered representative examples of modern systems with a wide range of applications in industry and transportation:

- Ballard Mark V (5 kW) – A highly efficient fuel cell designed for applications requiring reliability and long operational lifespan.
- BCS 500 W – A compact and flexible fuel cell suitable for smaller energy systems and experimental research.

The results were analyzed using two relevant statistical metrics: root mean square error (RMSE) and the sum of squared errors (SSE) to ensure an objective assessment of the proposed model’s accuracy. These indicators quantitatively evaluate the deviation between simulated and experimental data, clearly measuring the model’s precision. Notably, all proposed variants exhibit lower error values compared to all methods available in the literature. For the Ballard fuel cell, the best achieved RMSE value is 1.29631, while the corresponding SSE is 21.84576, demonstrating the model’s high accuracy in experimental data. Similarly, for the BCS 500 fuel cell, the best achieved RMSE is 0.10726, with a corresponding SSE of 0.20709, confirming that the proposed model reliably captures the performance of this type of fuel cell as well.

The low values of these indicators confirm the high precision of the proposed model, demonstrating an exceptionally strong agreement between simulated and experimental data. In addition to accuracy, a robust analysis of the model was conducted, including sensitivity testing to parameter variations and its applicability under different operating conditions, such as temperature and pressure fluctuations. These tests confirmed that the proposed method reliably describes the behavior of PEMFC fuel cells in real-world applications, which is crucial for their optimization and integration into energy systems.

The results of this study indicate that the combination of the SaDE algorithm and the *g*-function provides an effective solution for modeling and optimizing PEMFC fuel cells. Moreover, the proposed approach opens avenues for further research in the following areas:

- Experimental validation across a wider range of PEMFC fuel cells,
- Application of the model in fuel cell control and management systems,
- Further enhancement of optimization by integrating SaDE with other metaheuristic algorithms.

Therefore, the results of this study suggest that the proposed methodology can significantly contribute to a better understanding, modeling, and optimization of PEMFC fuel cells, thereby enhancing the performance of these promising technologies in the context of sustainable energy, transportation, and industrial applications. As an important direction for future work, particular attention will be given to hardware-in-the-loop (HIL) validation, in order to confirm the practical applicability of the proposed methodology under real operating conditions.

## Data Availability

The datasets used during the current study available from the corresponding author on reasonable request.

## Appendix

This appendix provides the technical specifications and experimentally measured polarization data (current–voltage characteristics) for the three commercial PEM fuel cell stacks used in this study: Ballard Mark, BCS, and NedStack PS6. In all datasets, *I*_*m*_ denotes the measured current (A), while *V*_*m*_ denotes the measured voltage (V) (Table A-1).

**Table A-1:** PEMFC technical documentation.

Parameter	Ballard Mark	BCS	NedStack PS6
Rated power (W)	5 000	500	6000
Voltage range (V)	19.6–33.3	17.3–29	37.38–61.64
Current range (A)	5.1–71.9	0.60–29.26	2.25–220.5
Max current density – Ma (A/cm²)	1.5	0.469	5
Number of cells - N	35	32	65
Active area (cm²)	50.6	64	240
Membrane thickness (µm)	178	178	178
Operating temperature (°C)	70	60	70
Hydrogen supply pressure (atm)	1	1	1
Oxygen supply pressure (atm)	1	0.2095	1

Ballard-Mark-V (5 kW) PEMFC.

Im=[0.10 0.21 0.32 0.40 0.55 0.68 0.74 0.85 0.95 1.11 1.21 1.33 1.42]*Ma;

Vm=[0.95 0.88 0.85 0.82 0.80 0.76 0.75 0.72 0.70 0.68 0.63 0.60 0.56]*N;

BCS (500 W) PEMFC.

Im=[0.60 2.10 3.58 5.08 7.17 9.55 11.35 12.54 13.73 15.73 17.02 19.11 21.20 23.00 25.08 27.17 28.06 29.26];

Vm=[29.00 26.31 25.09 24.25 23.37 22.57 22.06 21.75 21.45 21.09 20.68 20.22 19.76 19.36 18.86 18.27 17.95 17.30];

NedSTack PS6 (6 kW) PEMFC.

Im=[2.25, 6.75, 9, 15.75, 20.25, 24.75, 31.5, 36, 45, 51.75, 67.5, 72, 90, 99, 105.8, 110.3, 117, 126, 135, 141.8, 150.8, 162, 171, 182.3, 189, 195.8, 204.8, 211.5, 220.5];

Vm=[61.64, 59.57, 58.94, 57.54, 56.8, 56.13, 55.23, 54.66, 53.61, 52.86, 51.91, 51.22, 49.66, 49, 48.15, 47.52, 47.1, 46.48, 45.66, 44.85, 44.24, 42.45, 41.66, 40.68, 40.09, 39.51, 38.73, 38.15, 37.38];
